# *In vivo* Neuroregeneration to Treat Ischemic Stroke Through NeuroD1 AAV-Based Gene Therapy in Adult Non-human Primates

**DOI:** 10.3389/fcell.2020.590008

**Published:** 2020-11-05

**Authors:** Long-Jiao Ge, Fu-Han Yang, Wen Li, Tao Wang, Yu Lin, Jie Feng, Nan-Hui Chen, Min Jiang, Jian-Hong Wang, Xin-Tian Hu, Gong Chen

**Affiliations:** ^1^Key Laboratory of Animal Models and Human Disease Mechanisms of Chinese Academy of Sciences and Yunnan Province, Kunming Institute of Zoology, Chinese Academy of Sciences, Kunming, China; ^2^Kunming College of Life Science, University of the Chinese Academy of Sciences, Kunming, China; ^3^Guangdong-Hongkong-Macau Institute of CNS Regeneration, Jinan University, Guangzhou, China; ^4^National Resource Center for Non-Human Primates, Kunming Primate Research Center, and National Research Facility for Phenotypic and Genetic Analysis of Model Animals (Primate Facility), Kunming Institute of Zoology, Chinese Academy of Sciences, Kunming, China; ^5^State Key Laboratory of Medical Neurobiology and MOE Frontier Center for Brain Science, Institutes of Brain Science, Fudan University, Shanghai, China; ^6^CAS Center for Excellence in Brain Science, Chinese Academy of Sciences, Shanghai, China; ^7^Department of Biology, Huck Institutes of the Life Sciences, Pennsylvania State University, University Park, PA, United States

**Keywords:** *in vivo* reprogramming, cell conversion, astrocyte, neuron, non-human primate, ischemic stroke, brain repair, neuroregeneration

## Abstract

Stroke may cause severe death and disability but many clinical trials have failed in the past, partially because the lack of an effective method to regenerate new neurons after stroke. In this study, we report an *in vivo* neural regeneration approach through AAV NeuroD1-based gene therapy to repair damaged brains after ischemic stroke in adult non-human primates (NHPs). We demonstrate that ectopic expression of a neural transcription factor NeuroD1 in the reactive astrocytes after monkey cortical stroke can convert 90% of the infected astrocytes into neurons. Interestingly, astrocytes are not depleted in the NeuroD1-converted areas, consistent with the proliferative capability of astrocytes. Following ischemic stroke in monkey cortex, the NeuroD1-mediated astrocyte-to-neuron (AtN) conversion significantly increased local neuronal density, reduced microglia and macrophage, and surprisingly protected parvalbumin interneurons in the converted areas. Furthermore, the NeuroD1 gene therapy showed a broad time window in AtN conversion, from 10 to 30 days following ischemic stroke. The cortical astrocyte-converted neurons showed Tbr1^+^ cortical neuron identity, similar to our earlier findings in rodent animal models. Unexpectedly, NeuroD1 expression in converted neurons showed a significant decrease after 6 months of viral infection, indicating a downregulation of NeuroD1 after neuronal maturation in adult NHPs. These results suggest that *in vivo* cell conversion through NeuroD1-based gene therapy may be an effective approach to regenerate new neurons for tissue repair in adult primate brains.

## Introduction

Stroke is an important cause of long-term disability with a high mortality rate (Ovbiagele et al., 2013; Benjamin et al., 2018). Ischemic stroke is accounting for ∼87% of all strokes, and current treatments mainly target on the re-establishment of blood flow and neuroprotection (Avasarala, 2015). Stroke survivors often experience long-term disabilities due to a substantial loss of neuronal cells caused by ischemic injury. Therefore, in order to achieve better functional recovery after stroke, it is pivotal to regenerate new neurons after stroke to replenish the lost neurons and restore the lost brain functions.

Our group previously reported that *in vivo* overexpression of a single neural transcription factor NeuroD1 can convert endogenous glial cells directly into functional neurons (Guo et al., 2014), providing a new approach for neural repair in the brain and spinal cord. NeuroD1, neurogenic differentiation 1, is a bHLH transcription factor that plays an important role in neuronal differentiation. As one of the key neural transcriptional factors, NeuroD1 binds regulatory elements such as the promoters and enhancers of neuronal genes and drives neuronal fate determination (Pataskar et al., 2016). Our more recent studies also demonstrated that NeuroD1 AAV-based gene therapy has great potential in repairing the ischemic injured or stab lesioned mouse cortex through direct *in vivo* astrocyte-to-neuron conversion (Zhang et al., 2018; Chen et al., 2019). Besides NeuroD1, other groups have reported that expression of transcription factors Ngn2, Ascl1, Sox2, or combinations of factors can also convert internal glial cells into neurons in the brain or spinal cord (Grande et al., 2013; Niu et al., 2013, 2015; Su et al., 2014; Liu et al., 2015; Gascon et al., 2016; Jorstad et al., 2017; Pereira et al., 2017; Rivetti di Val Cervo et al., 2017). In addition to the overexpression of transcription factors, we have also demonstrated that small molecules can directly convert human glial cells into functional neurons (Zhang et al., 2015; Yin et al., 2019) through transcriptome-wide activation of neuronal genes (Ma et al., 2019). Other groups also reported direct chemical reprogramming of glial cells (Gao et al., 2017) or fibroblast cells (Ambasudhan et al., 2011; Hu et al., 2015; Li et al., 2015) into neurons. These studies point to the possibility of using brain internal glial cells to directly generate new neurons for brain repair. However, in order to test the therapeutic potential of *in vivo* cell conversion technology in human clinical trials, it is important to first understand in non-human primate (NHP) models besides rodent studies, because rodent brain is very different from human brain. Many experimental parameters obtained from rodent animal models cannot be extrapolated to human patients, evidenced by many failed stroke clinical trials over the past decades (O’Collins et al., 2006; Diener et al., 2008; Turner et al., 2013; Percie du Sert et al., 2017). This might be due to significant difference between rodents and primates in terms of brain volume, white matter content, complexity of brain structures, and genetic compositions. Large animal species may be more valuable animal models than rodents to evaluate the effectiveness of stroke therapy as they seem to closely mimic the human brain anatomy (Boltze et al., 2017; Modo et al., 2018).

Here, we provide direct evidence in Rhesus Macaque monkeys that expressing a single neural transcription factor NeuroD1 in reactive astrocytes caused by ischemic injury can convert them into neurons at the injury site. Following the *in situ* astrocyte-to-neuron (AtN) conversion, the neuronal density in the NeuroD1-treated injury areas were significantly increased, accompanied by an increase of dendritic marker and synaptic marker. Unexpectedly, parvalbumin interneurons were significantly protected from ischemic injury in NeuroD1-infected areas, while microglia and macrophage were significantly reduced after NeuroD1-treatment. Our findings suggest that *in vivo* cell conversion technology not only regenerate new neurons in the injury areas, but also ameliorate the microenvironment to be more neuro-protective and neuro-permissive.

## Results

### NeuroD1-Mediated Astrocyte-to-Neuron Conversion in the Monkey Cortex

We have previously demonstrated that reactive astrocytes inside mouse brains can be directly converted into functional neurons through expressing a single neural transcription factor NeuroD1 (Guo et al., 2014; Zhang et al., 2018; Chen et al., 2019). Because mouse brains are far different from human brains, it is uncertain whether such *in vivo* cell conversion technology would be applicable for future human clinical trials. To overcome this uncertainty, we decided to further investigate whether *in vivo* neuroregeneration approach can be reproduced successfully in adult NHP brains in order to pave the way toward future clinical therapies.

**FIGURE 1:**
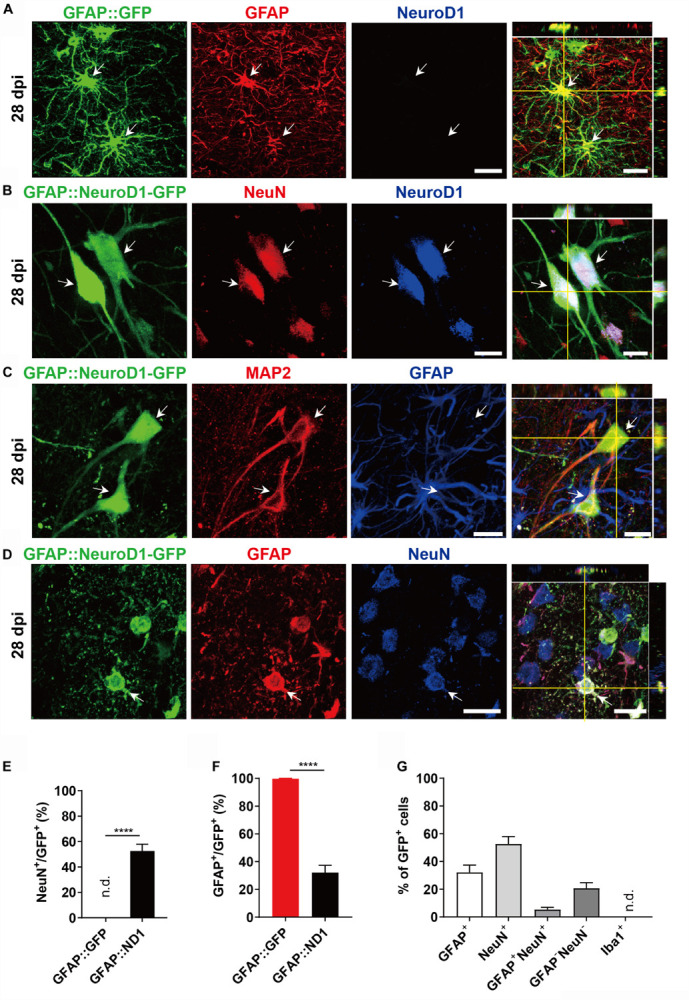
NeuroD1-mediated astrocytes-to-neuron conversion in the monkey cortex. **(A)** Injection of AAV9 viruses expressing GFP alone under human GFAP promoter (hGFAP::GFP) into monkey cerebral cortex infected astrocytes only with colocalization of astroglial marker GFAP (red) at 28 days post viral injection (dpi). These cells were negative for NeuroD1 staining. Scale bar, 20 μm. **(B,C)** Injection of AAV9 viruses expressing NeuroD1-GFP under human GFAP promoter (hGFAP::NeuroD1-P2A-GFP) converted astrocytes into neurons with colocalization of neuronal marker NeuN **(B)** and dendritic marker MAP2 **(C)**. The converted neurons (28 dpi) were confirmed to express NeuroD1 **(B)** but lost GFAP signal **(C)**. Scale bars, 20 μm. **(D)** Some hGFAP::NeuroD1-P2A-GFP infected cells (green) were immunopositive for both NeuN and GFAP, suggesting a transitional stage from astrocytes to neurons during the conversion process. Scale bar, 20 μm. **(E,F)** Cell counting analysis of NeuN^+^ or GFAP^+^ cells among viral infected cells. Data are presented as mean ± SEM. *N* = 30 random fields from triplicate sections in the control group; *N* = 40 random fields from triplicate sections in the ND1 group. n.d., not detected. Data presented as mean ± SEM. ****p < 0.0001 by Mann–Whitney test. **(G)** Bar graphs showing the different cell type compositions among all the NeuroD1-GFP infected cells. Data presented as mean ± SEM. *N* = 40 random fields from triplicate sections in the NeuroD1 side. n.d., not detected.

Adult Rhesus Macaque monkeys (male) aged from 9 to 21 years old were used in this study. AAV-based gene therapy was employed to test the efficacy of NeuroD1 (Chen et al., 2019) in converting the reactive astrocytes following ischemic cortical stroke into neurons in the monkey cortex. To infect the reactive astrocytes, we used astrocytic promoter GFAP to drive the expression of NeuroD1 or control reporter GFP. We first injected the control AAV9 GFAP::GFP viruses into the cortex of Rhesus Macaque monkey (monkey ID #04339) and found that the majority of GFP-infected cells were GFAP^+^ astrocytes at 28 days post viral infection (dpi), as expected (Figure 1A). In contrast, when injecting AAV9 GFAP::NeuroD1-GFP into the monkey cortex, we found that many NeuroD1-GFP-infected cells (both GFP^+^ and NeuroD1^+^) became NeuN^+^ neurons at 28 dpi (Figure 1B). Some NeuroD1-converted neurons also showed neuronal dendritic marker MAP2 (Figure 1C). Interestingly, like that reported in rodent models (Zhang et al., 2018; Chen et al., 2019), we found that some NeuroD1-GFP infected cells were immunopositive for both GFAP (red) and NeuN (blue) (Figure 1D), suggesting a transitional stage from astrocytes to neurons during the conversion process. Quantified data found that 99% of GFAP::GFP infected cells were GFAP^+^ astrocytes, but 52.6% of NeuroD1-GFP infected cells already became NeuN^+^ within 1 month of infection (Figures 1E,F). Among all the NeuroD1-GFP infected cells at 28 dpi, there were 26.8% GFAP^+^NeuN^–^ cells, 47.2% of GFAP^–^NeuN^+^ cells, and 5.4% of GFAP^+^NeuN^+^ cells together with 20.7% GFAP^–^NeuN^–^ cells (Figure 1G). These four groups represent the four subpopulations of cells infected by GFAP::NeuroD1-GFP. The GFAP^+^NeuN^–^ cells were non-converted astrocytes, and the GFAP^–^NeuN^+^ cells were converted neurons. The NeuN^+^/GFAP^+^ double positive cells and the NeuN^–^/GFAP^–^ double negative cells appeared to be transitional cells during AtN conversion process. Specifically, the NeuN^–^/GFAP^–^ cells might represent a population of NeuroD1-infected astrocytes that had lost GFAP signal but not yet acquired NeuN signal yet; whereas the NeuN^+^/GFAP^+^ cells after NeuroD1 infection might represent a population of newly converted neurons that did not lose GFAP signal yet but already acquired NeuN signal. These transitional cells were also observed during our rodent animal studies on NeuroD1-mediated AtN conversion (Guo et al., 2014; Zhang et al., 2018; Chen et al., 2019; Wu et al., 2020). These results suggest that overexpression of NeuroD1 in the reactive astrocytes of monkey brains can successfully convert astrocytes into neurons.

**FIGURE 2:**
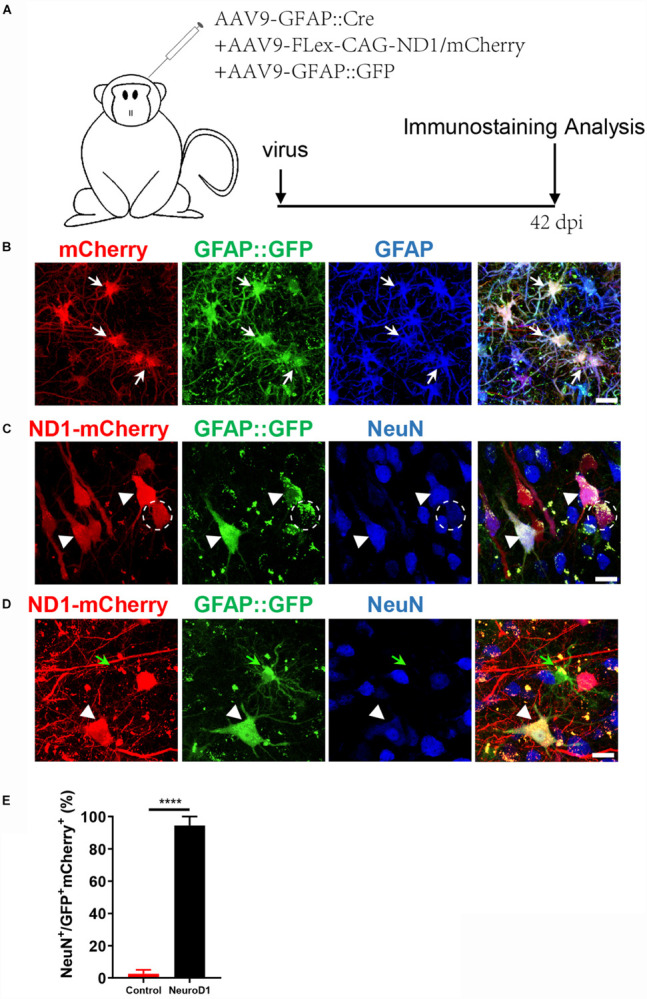
An engineered Cre-Flex system for high efficiency conversion in the monkey cortex. **(A)** Monkey cortex was injected with AAV9 hGFAP::GFP ^+^ GFAP::Cre together with either Flex-CAG::mCherry (control) or Flex-CAG::NeuroD1-mCherry and analyzed at 42 dpi. AAV9 GFAP::GFP was used to label local astrocytes in the cortex. **(B)** Representative images showing astrocytes (GFAP^+^) co-infected by the control viruses (AAV9 GFAP::GFP + GFAP::Cre + Flex-CAG::mCherry). Scar bars, 20 μm. **(C,D)** Representative images showing converted neurons (arrowhead, NeuN^+^) after infected by NeuroD1 viruses (AAV9 GFAP::GFP + GFAP::Cre + Flex-CAG::NeuroD1-mCherry) at 42 dpi. Note that GFAP::GFP-only infected cells were still astrocytes (**D**, green arrow). Scar bars, 20 μm. **(E)** Quantification of the neuronal conversion efficiency (NeuN^+^/GFP^+^mCherry^+^) among mCherry control (2.5 ± 2.5%) and NeuroD1-mCherry infected cells (94.4 ± 5.5%). The data are mean ± SEM. *N* = 9 regions from triplicate sections for each side. ****p < 0.0001 by Mann–Whitney test.

While GFAP promoter-driven NeuroD1-GFP expression converted astrocytes into neurons, some converted neurons might have lost GFP signal due to the reduced GFAP promoter activity during the neuronal conversion process, underestimating the actual conversion efficiency. To overcome this problem, we developed a Cre-FLEX system to separate GFAP promoter from NeuroD1 expression into two different AAV vectors (Chen et al., 2019). Specifically, Cre recombinase was controlled by GFAP promoter to target its expression in astrocytes (GFAP::Cre), while NeuroD1 expression was controlled by CAG promoter and flanked by LoxP sites in an inverted fashion (FLEX-CAG::NeuroD1-P2A-GFP or mCherry). To test the specificity of this Cre-FLEX system, we injected GFAP::GFP together with GFAP::Cre and FLEX-CAG::mCherry-P2A-mCherry or FLEX-CAG::NeuroD1-P2A-mCherry into the monkey cortex (Figure 2A). As expected, GFAP::GFP-infected cells were all GFAP-positive astrocytes (Figure 2B). Similarly, GFAP::Cre and FLEX-CAG::mCherry-P2A-mCherry also infected GFAP^+^ astrocytes, same as those infected by GFAP::GFP (Figure 2B). In contrast, when GFAP::GFP were injected together with GFAP::Cre and FLEX-CAG::NeuroD1-P2A-mCherry, many of the GFP and mCherry co-infected cells became NeuN^+^ neurons, suggesting successful conversion of astrocytes into neurons after NeuroD1 expression (Figure 2C, white arrowhead). Some NeuroD1-mCherry converted neurons showed much weaker GFP signal (Figure 2C, dashed circle), suggesting that the GFAP promoter activity of GFAP::GFP was downregulated. Note that the cells infected only by GFAP::GFP (no NeuroD1-mCherry) were still astrocytes (Figure 2D, green arrow), even though the neighboring astrocytes co-infected with NeuroD1-mCherry had become neurons (Figure 2D, white arrowhead). Quantitation of the NeuN^+^ cells among the viral infected cells by GFAP::GFP together with GFAP::Cre and Flex-NeuroD1-mCherry revealed high neuronal conversion efficiency (94.4 ± 5.5%) in the monkey cortex (Figure 2E). Together, these results demonstrate that AAV NeuroD1 Cre-FLEX system can successfully convert monkey cortical astrocytes into neurons, and the astrocyte-converted neurons can be labeled with reporters for long-term investigation.

### *In vivo* Neuroregeneration After Ischemic Injury in NHP Model

With the successful demonstration of NeuroD1-mediated astrocyte-to-neuron conversion in the monkey cortex, we next investigated whether such *in vivo* neuroregeneration technology can be used to repair damaged brains in NHPs. Recently, we have demonstrated that NeuroD1-based gene therapy can partially rescue motor functional deficits through converting reactive astrocytes into functional neurons in rodent ischemic stroke models induced by cortical injection of endothelin-1 (ET-1), a peptide that causes blood vessel constriction and hence ischemic injury (Chen et al., 2019). In accordance to our rodent studies, when injecting ET-1 into the motor cortex of monkeys, we also observed significant tissue damage at 3 weeks after ischemic injury (Supplementary Figure S1A). Specifically, compared to the non-stroke control cortex (Supplementary Figure S1A, top row), ET-1 injection caused significant tissue damage as revealed by immunostaining of NeuN, GFAP, and Iba1 at 3 weeks after focal ischemic stroke (Supplementary Figure S1A, bottom row). Note that, in ET-1 injected areas, NeuN significantly reduced across all injury areas, whereas GFAP signal also reduced in the lesion core areas but significantly increased in the peri-infarct areas, suggesting that astrocytes became reactive at 3 weeks following ischemic stroke. On the other hand, Iba1 staining showed significant increase in both the lesion core and the peri-infarct areas, suggesting a significant increase of neuroinflammation following ischemic stroke.

After establishing the focal ischemic injury model in the monkey cortex, we examined the effect of NeuroD1-mediated cell conversion on the injured cortical areas. We injected ET-1 (2 μg/μl; 2.5 μl each site; 6 sites for left cortex, with 5 mm apart between two sites, and 6 sites for right cortex) (Supplementary Figure S1B, red box) into both left side and right side of the monkey motor cortex to induce focal ischemic stroke. 3 weeks later, control mCherry AAV was injected into one side of the cortex and NeuroD1-mCherry AAV was injected into the other side of the cortex, both into the previous ET-1 injection sites. We then performed a series of immunostaining to investigate the neuronal and glial properties in the NeuroD1-infected cortical tissue versus the control mCherry-infected tissue at 2-month, 4-month, 6 months, and 1 year following a single dose of viral injection (Supplementary Figure S1B). Supplementary Figure S1C illustrates the gross morphology of monkey cortex after dissection at 2 months following viral injection. The control mCherry-injected site had visible cortical damage, whereas the NeuroD1-injected side showed much better tissue preservation (Supplementary Figure S1C, top row). This is confirmed by NeuN immunostaining (Supplementary Figure S1C, bottom row), which showed clear tissue loss in the mCherry-injected site but not NeuroD1-injected site. Similar results were also observed at other samples of 4 months and 1 year after viral injection (Supplementary Figures S1D,E). Together, ET-1 injection induces severe tissue loss in the monkey cortex, but much milder tissue damage after NeuroD1-treatment.

**FIGURE 3:**
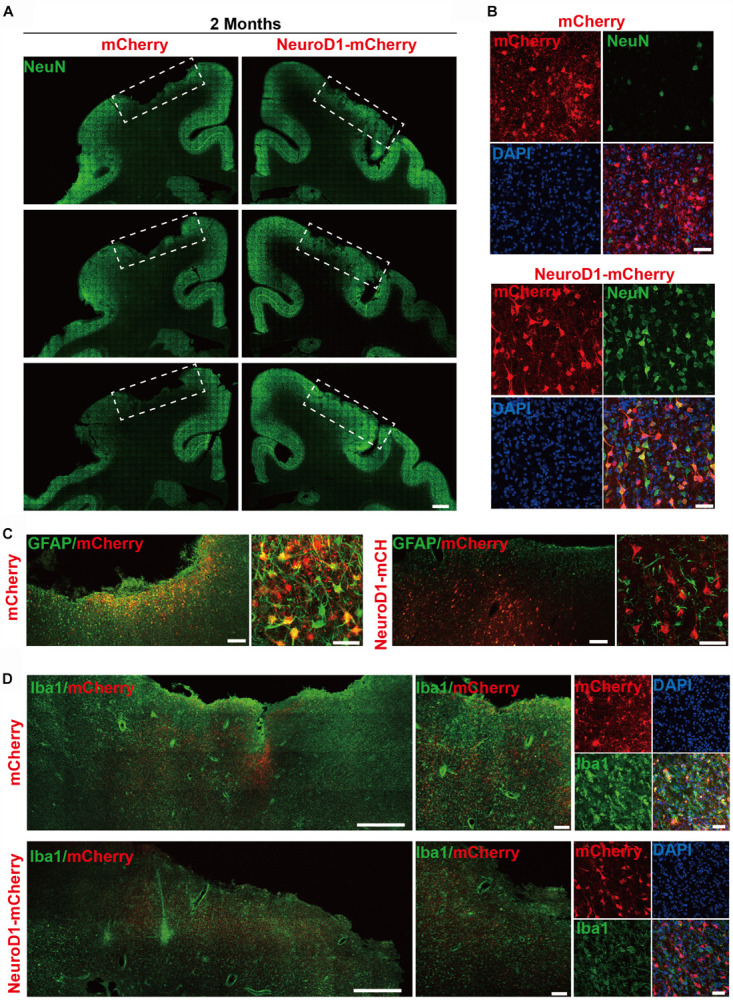
*In vivo* neuroregeneration and reduced inflammation after cell conversion in NHP ischemic stroke model. **(A)** Serial brain sections across the injury core illustrating the neuronal density (NeuN staining) in the monkey cortex following ischemic injury and viral injection. Note that both NeuN signal and cortical tissue were significantly impaired in the mCherry-injected side (left), but significantly rescued in the NeuroD1-mcherry injected side (right). Viral infection was conducted at 21 days post stroke (dps), and immunostaining analysis was performed at 2 months post viral injection (for all panels **A–D**). Scale bar, 2000 μm. **(B)** Representative high magnification images of neuronal density (NeuN, green) and viral infection (mCherry, red) in the monkey cortex. NeuroD1-mCherry infected areas always showed a significantly increased neuronal density. Nuclei were counterstained with DAPI (blue). Scale bar, 50 μm. **(C)** Both low and high magnification images illustrating the astrocytes (GFAP, green) infected by control virus mCherry alone (red, left panels), but rarely in the NeuroD1-mCherry infected areas (right panels). Note that while rarely co-localizing with NeuroD1-mCherry, GFAP^+^ astrocytes always persisted in the converted area and even showed less reactive morphology, indicating that astrocytes were not depleted after conversion. Scale bar, 200 μm (low mag), 20 μm (high mag). **(D)** Representative images in low and high magnification illustrate a reduction of microglia and macrophage (Iba1, green) in the NeuroD1-infected areas (bottom row) following ischemic stroke, comparing to the control side (top row). Nuclei are DAPI stained (blue). Scale bar, 1000 μm (low mag, left panels), 200 μm (higher mag, middle panels), 50 μm (highest mag, right panels).

**FIGURE 4:**
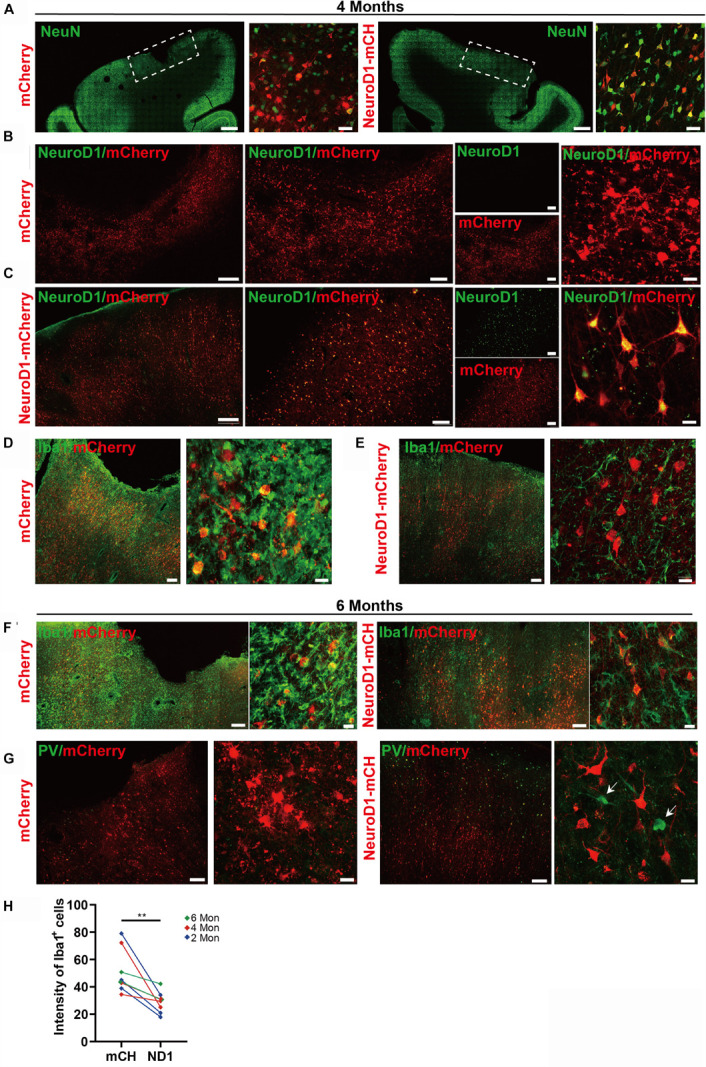
Long-term effect of NeuroD1-treatment in NHP ischemic stroke model. **(A)** Representative images illustrating neuronal density (NeuN, green), cortical tissue integrity, and viral infected cell morphology at 4 months post viral injection (virus injected at 21 dps, for panels **A–E**). Scale bars, 2000 μm for low mag, 50 μm for high mag. **(B,C)** Identification of NeuroD1 (green) expression at low and high magnification among viral infected cells. As expected, NeroD1 signal (green) was only detected in the NeuroD1-mCherry infected side **(C)**, but not the control side **(B)**. Note that the NeuroD1-mCherry infected cells displayed clear neuronal morphology. Scale bar, 500 μm (low mag, left 4 panels), 200 μm (higher mag, middle 4 panels), 20 μm (highest mag, right 2 panels). **(D,E)** Representative images in low and high magnifications illustrating significant reduction of microglia and macrophage (IBA1, green) in the NeuroD1-infected areas **(E)**, compared to the control mCherry infected areas **(D)**. Scale bar, 200 μm (low mag, middle panels), 20 μm (high mag, right panels). **(F)** Representative images illustrating a reduction of microglia and macrophage (IBA1, green) in NeuroD1-infected areas (right panels), compared to the control mCherry infected areas (left panels). Virus injected at 21 dps, and immunostaining performed at 6 months post viral injection (for both panel **F** and **G**). Scale bar, 200 μm (low mag), 20 μm (high mag). **(G)** Representative images illustrating the protection of parvalbumin (PV) interneurons (green, arrow) in and surrounding the NeuroD1-infected areas (right panels), compared to the control mCherry-infected areas (left panels). Scale bar, 200 μm (low mag), 20 μm (high mag). **(H)** Quantitation of the Iba1 intensity in the NeuroD1 side compared to the control side among the 8 monkeys injected with virus at 21 days following ischemic injury. **p = 0.0078 by Wilcoxon matched-pairs signed rank test.

To investigate the NeuroD1 treatment effect, we performed a series of immunostaining including NeuN, GFAP, and Iba1 to understand the overall neuronal and glial morphology in the stroke areas. Figure 3 illustrates one monkey (monkey ID #07041) at 2 months after viral infection. In the control side injected with mCherry alone (Figure 3A, serial sections in left column), we found a significant loss of NeuN signal as expected following ischemic injury. However, in the NeuroD1-mCherry injected side, NeuN staining showed much better NeuN signal in a serial section (Figure 3A, right column). Enlarged images in Figure 3B illustrate the significant difference in NeuN signal in the mCherry-infected areas (Figure 3B, top 4 panels) versus the NeuroD1-infected area (Figure 3B, bottom 4 panels). Note that most of the NeuroD1-mCherry infected cells were NeuN-positive, suggesting that many converted neurons contributed to the increased neuronal density in ischemically injured monkey cortex. GFAP immunostaining confirmed that while many mCherry-infected cells remained GFAP^+^ astrocytes in the control side (Figure 3C, left panels), the NeuroD1-mCherry infected cells were no longer astrocytes (lost GFAP signal) but showed neuronal morphology (Figure 3C, right panels). Furthermore, immunostaining with microglia and macrophage marker Iba1 also revealed that in comparison with the control mCherry-side (Figure 3D, top row), the NeuroD1-side showed a significant reduction of Iba1 signal (Figure 3D, bottom row). Similar results were also observed at 4 months (Figures 4A–E, monkey ID #060107) and 6 months (Figures 4F,G, monkey ID #04041) following viral infection. Specifically, at 4 months following viral infection, the NeuN signal in the control mCherry side was weaker than that in the NeuroD1-mCherry side (Figure 4A). The NeuroD1 signal was clearly detected in the NeuroD1-mCherry infected cells (Figure 4C, green signal) but not in the control side (Figure 4B). Immunostaining with Iba1 also revealed that compared to the mCherry-infected control areas (Figure 4D, green signal), there was a significant reduction of microglia and macrophage in the NeuroD1-mCherry infected areas (Figure 4E, green signal). At 6 months following viral infection, in comparison to the control mCherry side (Figure 4F, left panels), we also observed a reduction of Iba1-labeled microglia and macrophage (green signal) in the NeuroD1-mCherry infected areas (Figure 4F, right panels). Quantitative analysis of the Iba1 intensity found that the NeuroD1-treated side showed consistent decrease of Iba1 signal compared to the control side at 2, 4, and 6 months after viral infection (Figure 4H). Interestingly, when we investigated parvalbumin-positive (PV) GABAergic interneurons, which are often vulnerable after ischemic injury (Johansen et al., 1990; Tortosa and Ferrer, 1993; Wang, 2003; Povysheva et al., 2019), we found that many PV neurons were surprisingly protected in the vicinity of NeuroD1-mCherry infected areas (Figure 4G, right panels, comparing to the left control side). Together, these results suggest that NeuroD1-mediated *in vivo* AtN conversion not only generates new neurons in the ischemic injured areas in the monkey cortex, but also reduces microglia and macrophage and protects GABAergic neurons after ischemic injury.

### Increased Neuronal Density After NeuroD1-Treatment

**FIGURE 5:**
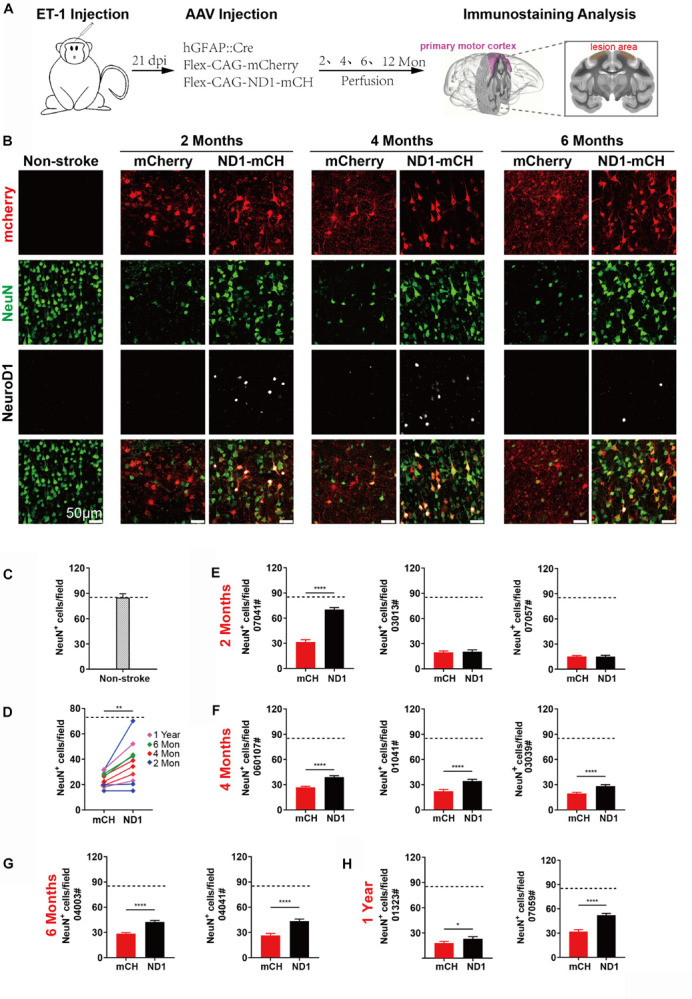
Increased neuronal density after NeuroD1-treatment. **(A)** Schematic illustration of our experimental design. **(B)** Representative images showing triple immunostaining of mCherry (red), NeuN (green) and NeuroD1 (white) in non-stroke cortex (left column) and the stroke cortex followed with viral injection (right 6 columns). NeuroD1-infected areas showed a consistent increase in the number of NeuN^+^ neurons (green) compared to the control side at 2, 4, and 6 months post viral infection. Note that NeuroD1 signal showed a significant decrease at 6 months compared to that at 2- and 4-months post viral infection. Scar bars, 50 μm. **(C)** Quantified data showing the mean number of NeuN^+^ cells in the motor cortex of non-stroke monkey. Data are represented as mean ± SEM. *N* = 30 random fields from triplicate slices. Each field = 0.1 mm^2^. **(D)** Quantitation of the neuronal density in the NeuroD1 side compared to the control side among the 10 monkeys injected with virus at 21 days following ischemic injury. **p = 0.0039 by Wilcoxon matched-pairs signed rank test. **(E–H)** Non-biased quantitative analyses on the neuronal density in the NeuroD1-treated versus control mCherry-treated cortex in each individual of the 10 monkeys. Viral infection was conducted at 21 days after ischemic injury, and immunostaining was performed at 2, 4, 6 months and 1-year post viral injection. Data are represented as mean ± SEM (*n* = 30–40 images per section, 6–8 sections per animal, total 180–240 fields/animal; Each field = 0.1 mm^2^). *p = 0.020, ****p < 0.0001 by Mann–Whitney test.

We next performed quantitative analysis on the neuronal density in the NeuroD1-treated versus mCherry control areas after ischemic injury. Figure 5A illustrates the experimental design, where AAV was injected at 21 days post ischemic injury and brain samples were collected at 2, 4, 6, and 12 months following viral infection. As shown in Figure 5B, when compared the NeuroD1-mCherry infected areas versus mCherry alone infected areas, the neuronal density (NeuN^+^ cells) in NeuroD1-infected areas was always higher than the mCherry-infected areas at 2, 4, and 6 months after viral infection (Figure 5B, 2nd row NeuN signal). Interestingly, NeuroD1 expression (Figure 5B, 3rd row) at 6-month time point appeared to be reduced comparing to the 2- and 4-month time points, which is worth of further investigation in future studies. To obtain a more general assessment of the injured cortical tissue beyond the NeuroD1-infected local regions, we adopted a non-biased quantitative analysis method by sampling the ischemic injured large cortical areas in both control and NeuroD1 sides. A total of 10 monkeys with ischemic injury on both sides of the motor cortex followed with viral injection (mCherry on one side, and NeuroD1 on the other side) were analyzed using the same method. To obtain accurate estimate of the neuron regeneration after NeuroD1-treatment, we took a systematic approach to perform immunostaining on 6–8 sections across the injury region (1000 μm interval) in the motor cortex of each monkey. For each section, 30–40 images across the injury regions were taken for quantification. Together, for each side of monkey M1 motor cortex, a matrix of ∼200 regions were sampled to cover the broad areas of viral infection for side-by-side comparison. Non-stroke cortical areas were also quantified in the same manner and yielded a neuronal density of 85/field (single section, each field area 0.1 mm^2^) (Figure 5C). Figure 5D illustrates the overall increase of neuronal density in the NeuroD1-infected cortices compared to the control virus-infected cortices among the 10 monkeys analyzed. The changes of neuronal density within each individual monkey were further illustrated in Figures 5E–H, from 2 months to 1 year of NeuroD1-treatment. Among 3 monkeys at 2 months post viral infection, one monkey (07041) showed a significant increase of neuronal density in the NeuroD1-treated side compared to the control side, while the other two monkeys did not show big difference between the two sides (Figure 5E). All three monkeys at 4-month time point (Figure 5F) and two monkeys at 6-month time point (Figure 5G) showed significant increase in the neuronal density after NeuroD1-treatment. After 1 year of viral infection, one monkey showed significant neuronal increase after NeuroD1-treatment while the other one only showed a mild increase (Figure 5H). The seemingly less recovery at 2-month time point might suggest that the neuronal recovery takes longer time in non-human primates than in rodents (Chen et al., 2019). Overall, among 10 monkeys treated with NeuroD1-based gene therapy, 7 monkeys showed a significant increase of neuronal density after a single dose of viral injection. Even for the 3 monkeys that did not show overall increase of neuronal density, the NeuroD1-infected local cortical areas typically still showed increase in the neuronal density (see Figure 5B for the NeuroD1-expressing regions).

If NeuroD1 converts many reactive astrocytes into neurons, it might raise a question whether such astrocyte-to-neuron conversion would deplete astrocytes in the converted areas. To address this concern, we performed GFAP immunostaining to examine astrocytes in the viral infected areas (Supplementary Figure S2). Compared to the ramified resting astrocytes (green signal) in the non-injured areas (Supplementary Figure S2A), the astrocytes in the injured areas following control viral injection showed highly hypertrophic morphology with astrocytic processes inter-tangled together (Supplementary Figures S2B–D, mCherry columns, green signal). In contrast, in NeuroD1-treated areas, GFAP-labeled astrocytes were always detected in the vicinity of NeuroD1-converted neurons at 2 months, 6 months, or 1 year after viral infection, and less reactive in morphology compared to the control group (Supplementary Figure S2B–D, NeuroD1-mCherry columns, green signal). Thus, NeuroD1-mediated high efficiency astrocyte-to-neuron conversion not only generates new neurons but also ameliorates reactive astrocytes without depleting local astrocytes, consistent with the intrinsic proliferative property of astrocytes.

### Regenerating Local Cortical Neurons After Ischemic Injury

**FIGURE 6:**
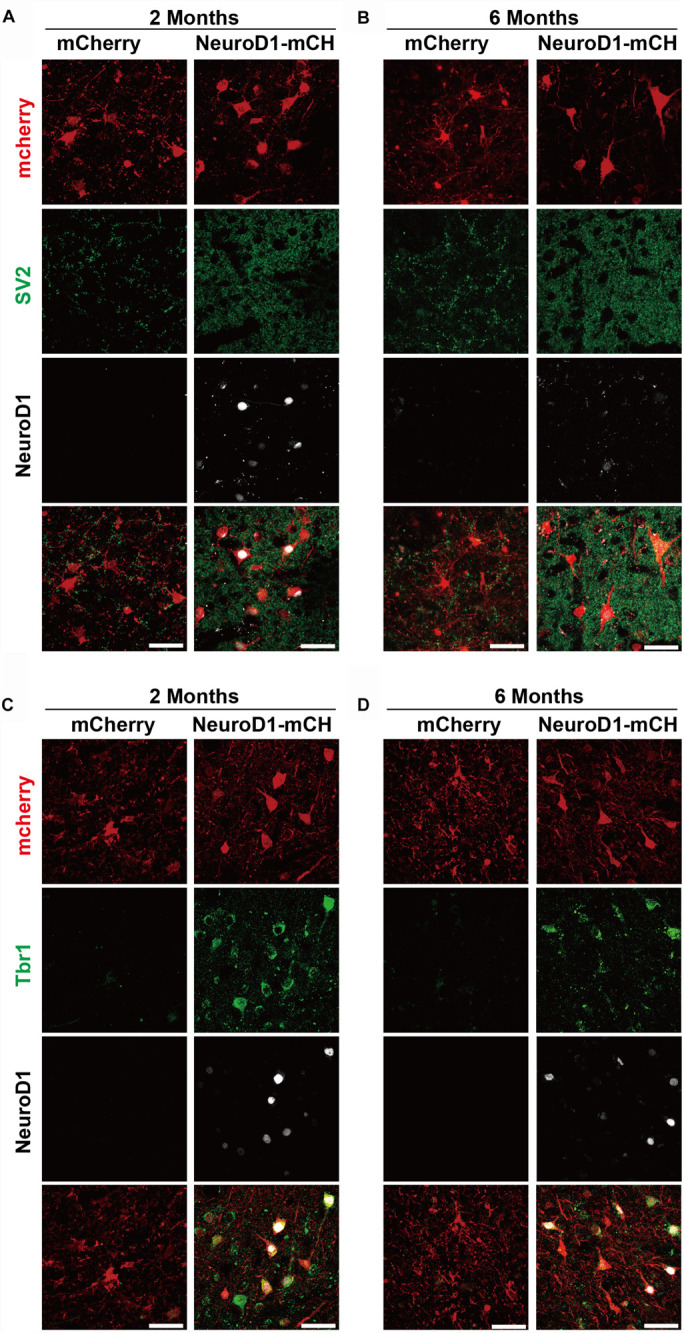
Cortical neuron identity for astrocyte-converted neurons in monkey cortex. **(A–B)** Representative images showing triple immunostaining of mCherry (red), SV2 (green) and NeuroD1 (white) in the NeuroD1-mCherry or mCherry-infected areas at 2- or 6-months post viral injection (21 days after stroke). Data shows significantly increased synaptic puncta (SV2) in the NeuroD1-infected areas, compared to the control mCherry-infected areas. Note a significant decrease of NeuroD1 expression at 6 months following viral infection. Scale bars, 50 μm. **(C,D)** Representative images showing triple immunostaining of mCherry (red), Tbr1 (green) and NeuroD1 (white) in the NeuroD1-mCherry or mCherry-infected areas at 2- or 6-months post viral injection (21 days after stroke). Most of the NeuroD1-expressing neurons were Tbr1^+^. Scar bars, 50 μm.

After the discovery of increased neuronal density following NeuroD1-treatment, we further examined synaptic marker SV2 in the viral infected areas. As shown in Figures 6A,B, at both 2- and 6-month time points following viral infection, the SV2 immunostaining showed a significant increase of puncta number in the NeuroD1-infected areas compared to the control mCherry-infected areas, consistent with better neuronal recovery after NeuroD1-treatment.

We next characterized the neuronal identity of the NeuroD1-converted neurons in ischemically injured monkey cortex. One difficulty of NHP studies is that many commercially available antibodies that work very well on rodent animals do not work for NHPs. After testing a series of cortical neuron markers, including Tbr1, CTIP2, and CUX2, we found that Tbr1 antibodies (Abcam, catalog #ab31940) showed positive signals in the Rhesus Macaque monkey brains. Tbr1 encodes a neuron-specific transcription factor of the T-box family and highly expressed in the mammalian cortex to affect the differentiation of projection neurons. Importantly, at both 2- and 6-month time points following viral infection, the NeuroD1-converted neurons showed immunoactivity for Tbr1 signal (Figures 6C,D), suggesting that NeuroD1-based gene therapy can regenerate cortical neurons in the monkey cortex after ischemic injury.

We further performed MAP2 immunostaining to assess the neuronal dendrites in the stroke areas after viral infection (Supplementary Figure S3). In normal cortex without injury, neuronal dendrites showed strong MAP2 signal (Supplementary Figure S3A). After stroke, MAP2 signal was largely lost in the control mCherry-infected areas (Supplementary Figures S3B,C, mCherry columns). However, in NeuroD1-infected areas, MAP2 was partially recovered at 2 months and significantly recovered at 6 months post viral infection (Supplementary Figures S3B,C, NeuroD1-mCherry columns). In supporting the immunostaining results, we also performed magnetic resonance imaging (MRI) analysis in one monkey at 1 year following ischemic stroke (Supplementary Figure S4). Consistent with immunostaining analyses, MRI analysis also revealed tissue loss in the mCherry-infected side (Supplementary Figure S4B, yellow arrow) but significant tissue preservation at the NeuroD1-infected side (Supplementary Figure S4B, black arrow). Note that the NeuroD1-infected areas did show some kind of tissue abnormality, indicating that it had experienced ischemic injury before. Together, these results suggest that AAV NeuroD1-based gene therapy can regenerate cortical neurons and repair cortical tissue following ischemic injury.

### Protection of GABAergic Neurons After Astrocyte-to-Neuron Conversion

**FIGURE 7:**
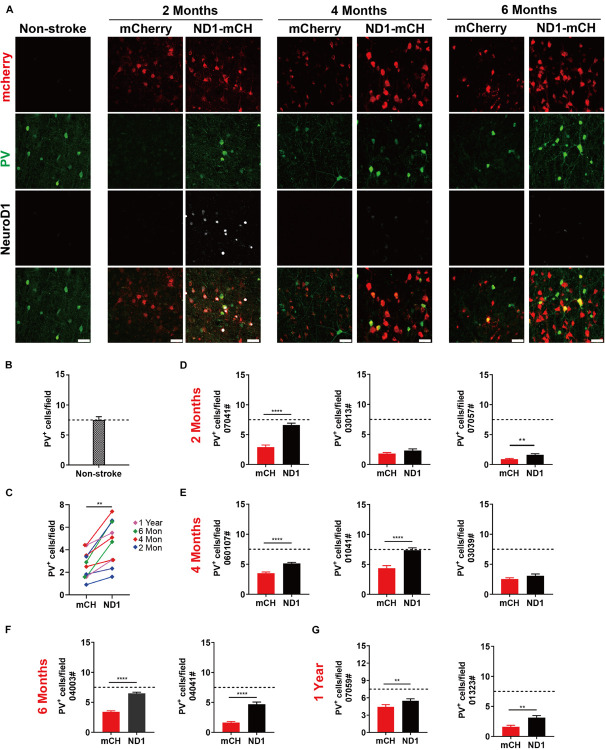
Protection of GABAergic neurons after astrocyte-to-neuron conversion. **(A)** Representative images showing triple immunostaining for mCherry (red), PV (green) and NeuroD1 (white) in non-stroke cortex (left column) and the NeuroD1-mCherry or mCherry-infected areas after stroke. Note a consistent increase of PV^+^ interneurons in NeuroD1-infected areas compared to the control mCherry-infected areas at 2, 4, and 6 months post viral infection (21 days after stroke). NeuroD1 expression decreased at 4 and 6 months after infection, but mCherry signal was still strong in the viral infected cells. Scar bars, 50 μm. **(B)** Quantified data showing the mean number of PV^+^ cells in the motor cortex of non-stroke monkey. Data are represented as mean ± SEM. *N* = 30 random fields from triplicate slices. Each field = 0.1 mm^2^. **(C)** Quantitation of the PV^+^ cell density in the NeuroD1-treated side compared with the control side among the 10 monkeys injected with virus at 21 days following ischemic injury. **p = 0.0020 by Wilcoxon matched-pairs signed rank test.**(D–G)** Quantitative analysis on the PV^+^ neuronal density in the NeuroD1-treated versus control mCherry-treated cortex in 10 monkeys. There were 6 out of 10 monkeys showed significant increase of PV^+^ neurons after NeuroD1-treatment. Viral injection at 21 days following ischemic injury, and immunostaining at 2, 4, 6 months and 1-year post viral injection. Data are represented as mean ± SEM (*n* = 180–240 fields/animal). **p < 0.01, ****p < 0.0001 by Mann–Whitney test.

As mentioned in Figure 4, we observed some PV neurons being protected in the NeuroD1-treated group (Figure 4G). It has been widely reported that ischemic injury tends to severely damage GABAergic neurons (Johansen et al., 1990; Tortosa and Ferrer, 1993; Wang, 2003; Povysheva et al., 2019). In accordance, we also found that compared to non-injured monkey cortex (Figure 7A, left column), ischemic injury caused a significant loss of PV signal in the mCherry control group (Figure 7A, mCherry columns, green signal). In contrast, in NeuroD1-treated side, many PV-positive neurons survived in the injured areas (Figure 7A, ND1-mCH columns, green signal). Interestingly, while the majority of NeuroD1-converted neurons were not PV^+^ GABAergic neurons, a small number of PV^+^ neurons in the NeuroD1-infected areas appeared to be mCherry^+^, consistent with our findings in the rodent animals that NeuroD1 overexpression can generate a small number of GABAergic neurons in the cortex (Guo et al., 2014). Quantitative analysis revealed a significant protection of PV neurons in the NeuroD1-treated side compared to the control mCherry sides. Among the 10 monkeys analyzed from 2 months to 1 year after viral infection, there was a significant increase in PV^+^ neurons in the NeuroD1-treated side compared to the control side (Figure 7C). Figures 7D–G illustrate the changes of PV^+^ neuronal density within each individual monkey, relative to the PV^+^ neuronal density in the non-stroke cortical areas (Figure 7B). The protection of GABAergic neurons after ischemic injury may be critical for preventing further brain damage such as potential epileptic seizures following ischemic stroke (Marco et al., 1997; DeFelipe, 1999; Camilo and Goldstein, 2004; Paz and Huguenard, 2015).

### Broad Time Windows of NeuroD1-Treatment After Ischemic Injury

**FIGURE 8:**
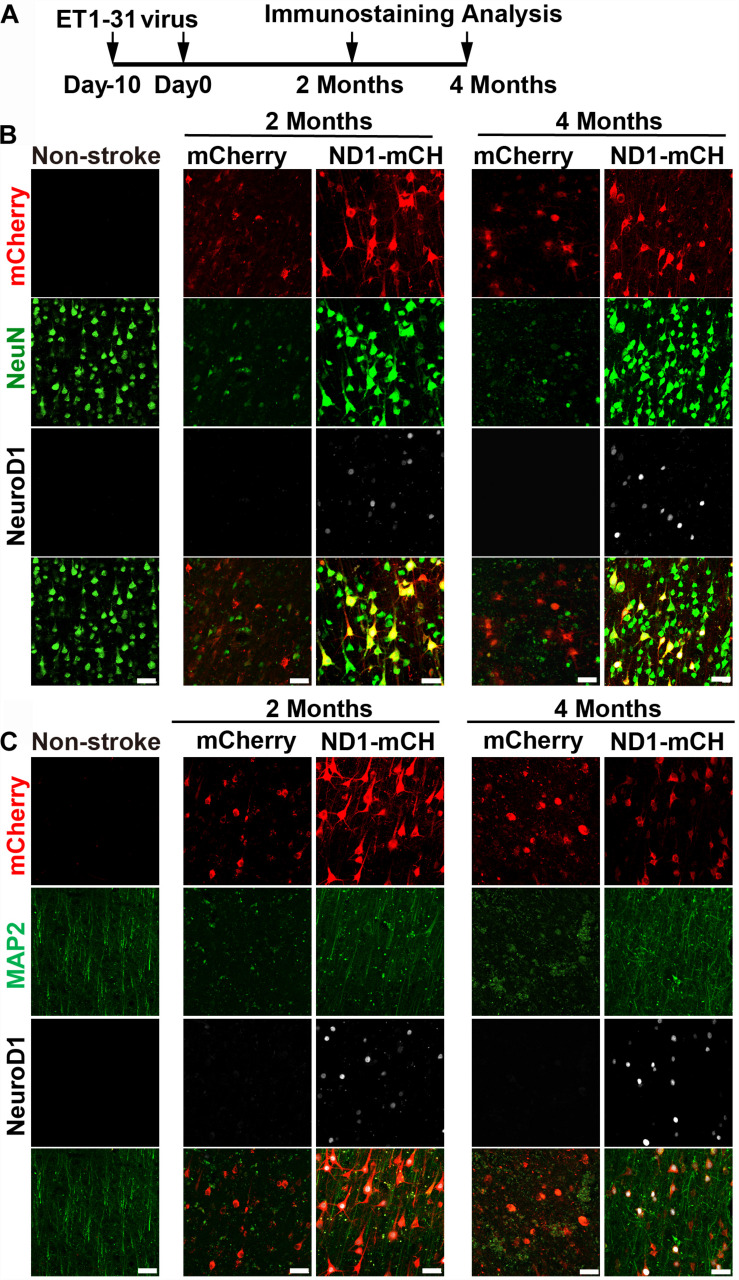
Beneficial effects of NeuroD1-based gene therapy delivered at 10 days after ischemic stroke in monkey cortex. **(A)** Experimental design for NeuroD1 treatment at 10 days after ischemic stroke. **(B)** Representative images showing mCherry (red), NeuN (green) and NeuroD1 (white) expression pattern in non-stroke cortex (left column) or in ischemic cortex after virus infection (right 4 columns). NeuroD1-infected areas consistently showed higher neuronal density. Scar bars, 50 μm. **(C)** Representative images showing mCherry (red), MAP2 (green), and NeuroD1 (white) expression pattern in non-stroke cortex (left column) or in ischemic cortex after viral injection (right 4 columns). The neuronal dendritic marker MAP2 signal increased significantly in NeuroD1-infected areas, compared to the control mCherry-infected areas. Scar bars, 50 μm.

Finally, we injected NeuroD1 AAV at different time windows following ischemic injury. Besides viral injection at 21 days after stroke in 10 monkeys shown above, we also injected mCherry control or NeuroD1-mCherry AAV at 10 days post ischemic injury (10 dpi) (Figure 8 and Supplementary Figure S5) or 30 days post ischemic injury (Supplementary Figures S6, S7). Figure 8A illustrates the experimental design on two monkeys injected AAV at 10 days post stroke. Similar to the experiments shown above for viral injection at 21 days post stroke, we consistently observed increased neuronal density (Figure 8B, NeuN staining) and better recovery of MAP2 signal in the NeuroD1-infected areas (Figure 8C, green signal) at both 2- and 4-month time points following viral infection. Furthermore, the NeuroD1-infected areas also showed less reactive microglia and macrophage (Supplementary Figure S5A) and more preserved PV-positive neurons (Supplementary Figure S5B). The same results were also observed in two monkeys injected AAV at 30 days following ischemic stroke (Supplementary Figure S6A). Specifically, NeuroD1-treatment showed significant increase in neuronal density (Supplementary Figure S6B, NeuN signal in green), better recovery in MAP2-labeled dendrites (Supplementary Figure S6C, green signal), less reactive microglia and macrophage (Supplementary Figure S7A), and more preserved PV-positive neurons (Supplementary Figure S7B). While more experiments are necessary to test the optimal time window, our results suggest that NeuroD1 AAV-based gene therapy may have a broad treatment window ranging from 10 to 30 days post ischemic stroke to have effective therapeutic intervention.

## Discussion

Stroke is afflicting millions of patients worldwide but few clinical trials have succeeded in the past (O’Collins et al., 2006; Diener et al., 2008; Turner et al., 2013; Percie du Sert et al., 2017). As the first proof-of-concept study in NHPs to test *in vivo* cell conversion technology, we employed a focal stroke model through intracranial injection of endothelin-1 (ET-1) to induce blood vessel constriction in the motor cortex of Rhesus Macaque monkeys. Although the middle cerebral artery occlusion (MCAO) model is widely used in rodent animals, previous studies in NHPs reported high mortality rate of MCAO model and large variations in the infarct size (Cook and Tymianski, 2012). In contrast, we had established successful ET-1-induced focal stroke model with very low mortality rate and consistent cortical damage in rodents (Chen et al., 2019). In this study, we demonstrate that ectopic expression of a single neural transcription factor NeuroD1 in the reactive astrocytes of monkey cortex after ischemic stroke can successfully convert astrocytes into neurons. This NeuroD1-mediated AtN conversion technology is built upon our earlier success in *in vivo* rodent animal models as well as *in vitro* human astrocyte cultures (Guo et al., 2014; Zhang et al., 2018; Chen et al., 2019; Wu et al., 2020), suggesting a broad application of such AtN conversion technology across different species for brain repair. Successful applications of our NeuroD1 AAV-based gene therapy in NHP models might bridge some gaps between rodent models and future clinical trials.

### Advantages of Using Large Animal Models for Evaluating Therapeutic Treatment After Stroke

Compared to small lissencephalic rodent brains, large animal models such as monkeys used here are closer to humans because they have larger brain volume, higher ratio of white matter to gray matter, and more complexity of glial cells than rodents (Modo et al., 2018; Herrmann et al., 2019). Previous studies also reported stem cell therapy using large animals such as sheep for stroke repair (Werner et al., 2015; Boltze et al., 2008, 2019). In the past, stem cell therapy has been extensively explored as a cell replacement therapy to treat stroke, but it has long suffered from low survival rate of the transplanted cells after long-term engraftment. Recent advancement suggested that stem cell therapy might modulate immune responses and can be combined with other therapeutic interventions such as rehabilitation and recanalization to enhance the efficacy (Modo et al., 2018; Boltze et al., 2019; Herrmann et al., 2019). Compared to stem cell therapy that relies upon transplantation of external cells, our gene therapy treatment is more relying upon internal glial cells to regenerate new neurons that can survive more than 1 year in the NHP brains as shown here. Such long-term neuronal survival may offer the possibility to restore damaged neural circuits after ischemic injury or other types of brain injury. Of course, we can also envision that our neuroregenerative gene therapy can be further combined with other strategies such as recanalization, rehabilitation, pharmacological intervention, or even stem cell therapy for additive or synergistic benefits (Jovin et al., 2016; Savitz et al., 2019).

### Comparison of *in vivo* Cell Conversion Between NHP and Rodent Models

Neuronal loss is the most common cause of brain functional deficits after acute injury such as stroke and concussion as well as chronic neurodegenerative disorders such as Alzheimer’s, Parkinson’s and Huntington’s disease. Neuroregeneration in adult mammalian CNS has been proved to be one of the most difficult tasks in the entire regenerative medicine field, largely because neurons cannot divide to regenerate themselves and external cell transplantation yields very low number of functional new neurons (Goldman, 2016). To overcome the limitations of cell transplantation therapy, we and other research groups have developed *in vivo* cell conversion technology to regenerate functional new neurons from endogenous glial cells for brain repair (Guo et al., 2014; Liu et al., 2015; Niu et al., 2015, 2018; Torper et al., 2015; Gascon et al., 2016; Wang et al., 2016; Pereira et al., 2017; Karow et al., 2018; Zhang et al., 2018; Chen et al., 2019). While stab injury model has been commonly used in various *in vivo* cell conversion studies, we have previously demonstrated in a mouse Alzheimer’s disease model that NeuroD1 can convert reactive astrocytes into functional neurons in 14-month old AD mouse brains (Guo et al., 2014). Recently, we have further demonstrated in a mouse stroke model that NeuroD1-based gene therapy can successfully convert reactive astrocytes into functional neurons and promote functional recovery (Chen et al., 2019). By combining NeuroD1 and Dlx2 together, we further demonstrated that astrocytes in the striatum of mouse model with Huntington’s disease can be directly converted in medium spinal neurons, which not only improved motor functions but also extended the life span of HD mice (Wu et al., 2020). Other group also reported that striatal astrocytes can be converted into dopaminergic neurons and promote behavioral recovery in a mouse PD model (Rivetti di Val Cervo et al., 2017). These successful *in vivo* cell conversion studies in various rodent animal models suggest a great potential in neuroregeneration and neural repair. Nevertheless, since so many clinical trials on CNS disorders have failed in the past based on successful preclinical studies in rodent animal data, we believe that it is essential to establish solid foundation in NHP models before starting clinical translation for human brain repair. Some successful discoveries made in the rodent animal models may not be applicable to primates. For example, a recent study generated a new serotype of AAV PHP.eb that showed remarkable permeability through blood-brain-barrier in mice and could infect essentially the entire brain with high efficiency (Chan et al., 2017). However, it was later found that such AAV serotype was better suited for rodent animals only, and did not show high infection efficiency in NHPs (Liguore et al., 2019), suggesting that it would be a disaster should someone jump from mouse success directly to human clinical trials using this viral serotype. For stroke studies, it is also known that the blood vessel branching network and lateral circulation in rodents are very different from primates (Masakatsu et al., 2003; Shunichi and Del Zoppo, 2003; Shinichi et al., 2004; Cuccione et al., 2016; Du et al., 2016). Therefore, the ischemic injury and its post-injury compensation may well be different between rodents and primates. Our current study extends our previous success on *in vivo* AtN conversion achieved in rodent animals (Guo et al., 2014; Zhang et al., 2018; Chen et al., 2019; Liu et al., 2020; Wu et al., 2020), and provides direct evidence that NeuroD1-based gene therapy can convert reactive astrocytes into neurons in monkey brains suffered from ischemic injury. This *in vivo* NHP model fills the gap between *in vivo* rodent models and *in vitro* human glial cell culture models with regard to the translational potential of AtN conversion technology. Indeed, current gene therapy products on the market including Luxturna from Spark Therapeutics and Avexis ZOLGENSMA have both obtained successful NHP data (Jacobson et al., 2006; Towne et al., 2010; Passini et al., 2014) before conducting human clinical trials.

One unexpected observation in our NHP study on NeuroD1-mediated AtN conversion is the gradual decrease of NeuroD1 expression level at 6 months after viral infection, which was not observed in mice, highlighting the difference between rodents and primates. As a neural transcription factor, NeuroD1 is known to be expressed in a subset of neural stem cells during early brain development and then decrease in the adult brain (Pleasure et al., 2000; Cho and Tsai, 2004; Gaudillière et al., 2004; Lee et al., 2010; MuñOz et al., 2010; Pataskar et al., 2016), except the adult hippocampus in mice (Gao et al., 2009; Tomoko et al., 2009). In both mouse and monkey brains, immunostaining with NeuroD1 antibodies always detected very low level of NeuroD1 signal in the cortical neurons. In NeuroD1-infected cells, NeuroD1 signal is typically very high compared to the non-infected cells. This is true in monkey brains at 2- and 4-month time points after viral infection, but we observed a significant decrease in NeuroD1 signal within many NeuroD1-mCherry infected cells at 6-month time point. While the precise mechanism of such decrease of NeuroD1 expression after long-term neuronal conversion and maturation requires further investigation, it suggests that the NeuroD1 expression from the AAV episome may be significantly downregulated in mature neurons, for example through phosphorylation-regulated silencing mechanism. Such spontaneous downregulation of NeuroD1 after neuronal conversion might be an advantage for future clinical trials, because NeuroD1 may not stay at a high level in converted mature neurons.

It is worth to emphasize that there are also many similarities between rodent and NHP models regarding AtN conversion. One of the most consistent findings is that ectopic expression of NeuroD1 in cortical reactive astrocytes after ischemic injury can efficiently convert astrocytes into neurons (90% conversion efficiency) regardless of rodents or NHPs. Another interesting finding is that, while white matter occupies much larger area in monkey brains, we rarely detect any converted neurons in the monkey white matter, which is consistent with our recent findings in rodent white matter (Liu et al., 2020).

Because AAV can infect both neurons and glial cells in the mammalian brains, one has to be cautious that the newly generated neurons are indeed converted from glial cells, not due to the infection of pre-existing neurons. In particular, when we developed Cre-FLEX AAV system, we did observe some neurons were infected by our control viruses GFAP::Cre + FLEX-mCherry (Figure 2E, mCherry control). Similar findings were also reported in our recently published work in both rodent stroke model (Chen et al., 2019) and HD model (Wu et al., 2020), that a small percentage of neurons were infected by control AAV expressing GFP or mCherry alone. These AAV-infected neurons in the control group could be caused by intercellular trafficking of extracellular vesicles carrying Cre from AAV-infected astrocytes to the surrounding neurons, since such extracellular vesicle trafficking has been reported before (Budnik et al., 2016; Venturini et al., 2019). To test this possibility, we have further performed Cre immunostaining in monkey brain slices infected by AAV. As shown in Supplementary Figure S8, Cre signal was detected clearly in the GFAP^+^ astrocytes in the control group (GFAP::Cre and FLEX-mCherry) at 2 weeks, 2 months, and 6 months after viral infection (Supplementary Figures S8A–C, left side). In contrast, in NeuroD1 group, Cre signal was only detected at early time of infection at 2 weeks in GFAP-positive astrocytes but not at 2–6 months after viral infection (Supplementary Figures S8A’–C’, right side), confirming that astrocytes have been converted into neurons and the GFAP promoter activity of GFAP::Cre has been downregulated after neuronal conversion. The significantly reduced Cre signal in the NeuroD1 group is in sharp contrast to the high expression level of Cre signal in the control group, suggesting that the potential leakage of Cre signal out of NeuroD1-infected cells is much lower than the control mCherry-infected cells. To further demonstrate that our Cre-FLEX system can convert dividing astrocytes into neurons, we performed BrdU labeling experiment due to the fact that BrdU can only be incorporated into the DNA during cell proliferation. Since neurons cannot divide, such BrdU experiment will provide further evidence for cell conversion from dividing glial cells. To save the usage of monkeys for this single experiment, we performed BrdU experiment in mice instead (Supplementary Figure S9). BrdU was injected after stroke for 5 consecutive days to label dividing glial cells before injecting AAV GFAP::Cre and FLEX-mCherry or FLEX-NeuroD1-mCherry viruses. After 2 weeks of viral infection, we detected a significant number of BrdU-labeled neurons in the NeuroD1 group, suggesting that they were indeed converted from dividing astrocytes. Together, while we cannot exclude the possibility of intercellular trafficking of GFAP::Cre from AAV-infected astrocytes to surrounding neurons, the majority of neurons generated in the NeuroD1 group are converted from astrocytes, as demonstrated clearly by AAV GFAP::NeuroD1 vectors.

### Broad Time Window for NeuroD1 AAV-Based Gene Therapy

Previously, there have been a number of stroke studies in NHP models but they are mainly testing drug effects in acute stroke model typically within a few hours after stroke (Takamatsu et al., 2001; Yasuhisa et al., 2003; Cook et al., 2012, 2017). In our current study, we have tested NeuroD1-mediated neuronal conversion at three different time windows: 10 days post stroke (3 monkeys); 21 days post stroke (10 monkeys); and 30 days post stroke (3 monkeys). At all these 3 time points, we found that reactive astrocytes were converted into neurons by AAV NeuroD1. The fact that neurons can be regenerated at least 1 month following stroke suggests that our *in vivo* cell conversion technology may have a broad time window for stroke treatment. Furthermore, the neuronal recovery after NeuroD1 treatment is long lasting, ranging from 2 months to 1 year after a single dose of AAV treatment. Such structural improvement at the cellular level may be critical for further functional improvement, which will be tested in ongoing studies.

Interestingly, similar to our recent observations made in mouse stroke studies (Chen et al., 2019), we also observed significant protection of interneurons after NeuroD1 treatment in monkey stroke model. It is known that interneurons are vulnerable under ischemic injury (Johansen et al., 1990; Tortosa and Ferrer, 1993; Wang, 2003; Povysheva et al., 2019). The neuroprotective effect of *in vivo* cell conversion mediated by NeuroD1 may have important implications for therapeutic treatment. The precise mechanisms are to be investigated in further studies, but our observation of reduced reactive microglia and macrophage at the NeuroD1-infected areas may at least contribute to the protection of interneurons as well as other neurons.

It is worth to mention that one big challenge about working on non-human primates is that many antibodies that work very well for rodent animals often do not work for monkeys. To identify the variety of neuronal subtypes in monkey brains, it is necessary to apply new technologies such as single cell sequencing technology to better characterize the identity of newly converted neurons.

### Perspective on NeuroD1 AAV as a Potential Therapeutic Agent for Neuroregenerative Gene Therapy

Many previous clinical trials on stroke repair have not been successfully translated from preclinical studies in rodents (Turner et al., 2013). A successful demonstration of neural repair in adult NHP models after stroke may be an important step toward potential translation into clinical therapies. Our current study in NHP stroke model, together with our recent rodent stroke study (Chen et al., 2019), shows a consistent finding that NeuroD1-based gene therapy can regenerate a large number of new neurons in the adult mammalian brains. The limitation of this current NHP study is that the neuronal recovery at the cellular level needs to be further correlated with behavioral improvement, which requires further studies using sophisticated motor functional tests that can monitor long-term functional deficits after stroke. Another limitation is that the current stroke model is a focal stroke model, with limited total infarct volume caused by stroke. Further studies will test more severe stroke models such as cerebral artery occlusion or hemorrhagic stroke models in NHPs to better mimic patient conditions in order to develop more suitable therapeutic interventions for patients. The third limitation is small sample size at any specific time point because it is difficult to use many NHPs for pathological analyses. This first study covered a broad time window from 2 months to 1 year after viral infection to evaluate the cell conversion effect. Our next project can overcome this sample size limitation by focusing on a specific time point. In addition, for future studies, we will also employ more sophisticated imaging techniques, such as magnetic resonance imaging (MRI) or PET scan to better assess the lesion size and even potential local neuronal activity after conversion to evaluate the longitudinal effect of our neuroregenerative gene therapy in nonhuman primate models (Werner et al., 2015; Boltze et al., 2019).

## Materials and Methods

### Animal Preparation

Eighteen adult rhesus macaques (*Macaca mulatta*), aged from 9 to 21 years old (mean body weight = 8.5 kg), were employed in this study. The monkeys were provided by the Kunming Primate Research Center, Kunming Institute of Zoology, Chinese Academy of Sciences, China. All rhesus macaques were male and healthy without history of neurological diseases, and were housed in individual cages with a standardized light/dark cycle and were fed under standard procedures. All experimental procedures and animal care were approved by the Ethics Committee of Kunming Institute of Zoology and the Kunming Primate Research Center (approval NO. IACUC19007), Chinese Academy of Sciences, performed in accordance with National Institutes of Health guidelines for the care and use of laboratory animals. The animal care facility was accredited by the Association for Assessment and Accreditation of Laboratory Animal Care (AAALAC). One monkey (ID #04013) was used in the preliminary study for the establishment of focal ischemic stroke model, and another monkey (ID #04339) was tested for *in vivo* astrocyte-to-neuron conversion without stroke. Other sixteen monkeys were used for focal ischemic stroke followed by viral injection. To use minimal numbers of monkeys, one side of the monkey cortex was used for control viral injection, and the other side of monkey cortex was injected with NeuroD1 AAV for cell conversion test. Information for these monkeys was listed in Table 1.

**TABLE 1:** Animal summary.

Animals	Sex	Age	Bodyweight (KG)	ET1 injury	Virus	Virus Duration
04339#	M	12	6.7	——	AAV-GFAP::Cre+AAV-Flex-CAG-ND1/mCherry+ AAV-GFAP::GFP; GFAP::GFP; GFAP::ND1-GFP	4–6 weeks
04013#	M	12	8.6	3 weeks	——	——
07057#	M	9	12.0	3 weeks	AAV-GFAP::Cre+AAV-Flex-CAG-ND1/mCherry	2 Month
07041#	M	9	10.4	3 weeks	AAV-GFAP::Cre+AAV-Flex-CAG-ND1/mCherry	2 Month
03013#	M	10	12.5	3 weeks	AAV-GFAP::Cre+AAV-Flex-CAG-ND1/mCherry	2 Month
01041#	M	15	6.7	3 weeks	AAV-GFAP::Cre+AAV-Flex-CAG-ND1/mCherry	4 Month
060107#	M	10	7.1	3 weeks	AAV-GFAP::Cre+AAV-Flex-CAG-ND1/mCherry	4 Month
03039#	M	13	11.0	3 weeks	AAV-GFAP::Cre+AAV-Flex-CAG-ND1/mCherry	4 Month
04003#	M	12	9.6	3 weeks	AAV-GFAP::Cre+AAV-Flex-CAG-ND1/mCherry	6 Month
04041#	M	12	10.1	3 weeks	AAV-GFAP::Cre+AAV-Flex-CAG-ND1/mCherry	6 Month
07059#	M	9	8.5	3 weeks	AAV-GFAP::Cre+AAV-Flex-CAG-ND1/mCherry	1 year
01323#	M	15	16.0	3 weeks	AAV-GFAP::Cre+AAV-Flex-CAG-ND1/mCherry	1 year
03021#	M	14	7.1	10 days	AAV-GFAP::Cre+AAV-Flex-CAG-ND1/mCherry	2 Month
01073#	M	16	6.9	10 days	AAV-GFAP::Cre+AAV-Flex-CAG-ND1/mCherry	2 Month
05025#	M	12	8.7	10 days	AAV-GFAP::Cre+AAV-Flex-CAG-ND1/mCherry	4 Month
07089#	M	10	7.7	30 days	AAV-GFAP::Cre+AAV-Flex-CAG-ND1/mCherry	2 Month
04307#	M	13	7.1	30 days	AAV-GFAP::Cre+AAV-Flex-CAG-ND1/mCherry	2 Month
96079#	M	21	8.0	30 days	AAV-GFAP::Cre+AAV-Flex-CAG-ND1/mCherry	4 Month

### Stroke Model

Food, but not water, was deprived in the evening prior to the surgery day to avoid potential choke of food during anesthesia. Anesthesia was induced with hydrochloric acidulated ketamine (10 mg/kg, intramuscular injection) and maintained with sodium pentobarbital (Merck; 20 mg/kg, i.m.). Atropine (0.02 mg/kg, i.m) was given preoperatively to decrease secretions. After anesthesia, the pain reflex was checked before surgery operation. Local lidocaine was applied before the operation to further reduce pain. For cortical injection, the monkey was placed on a warm heated surgical table and mounted on a stereotaxic apparatus (68912, Shenzhen Reward Life Science, Shenzhen, China), which has a resolution of 100 micrometer (0.1 mm accuracy) in terms of X-Y horizontal movement. The Z-axis movement of the injecting needle was controlled by a microsyringe pump (UMP3-2, Micro4, WPI Apparatus, Sarasota, United States), with a resolution of 10 micrometer. The focal ischemic lesion was created in the bilateral primary motor cortex (M1) area. The animals were operated with a midline scalp incision followed by drilling holes on the skulls above motor cortex. Micro-injection of Endothelin-1 (1-31) (Human) (Peptide, 4360-s, Japan) was made at six sites in each side of the M1 area to induce focal ischemic injury according to a combined MRI and Histology Atlas of the Rhesus Monkey Brain in Stereotaxic Coordinates (2nd Edition, Kadharbatcha S. Saleem, Nikos K. Logothetis, 2012) at the following coordinates: (1) anteroposterior (AP), 18.8 mm; mediolateral (ML), 11.2 mm, (2) AP, 17.3 mm; ML, 13.5 mm, (3) AP, 15.7 mm; ML, 16.1 mm, (4) AP, 16.6 mm; ML, 8.9 mm, (5) AP, 15.6 mm; ML, 11.1 mm, (6) AP, 14.4 mm; ML, 13.9 mm. A volume of 2.5 μL (2 μg/μL) of ET-1 was injected at each site using a 10-μL Hamilton syringe and infusion pump (UMP3-2, Micro4, WPI Apparatus, Sarasota, United States) at a speed of 100 nl per minute. Six injections were given 4 mm under the dura of monkey. Needle was inserted 4 mm under the dura and left in place for 5 min to allow the needle completely fit into brain tissues. Then the needle was withdrawn to 2 mm under the dura, and started to inject after 2-min pause. ET-1 was injected at each site in five boluses of 500 nL, allowing a 2-min pause and 500 μm distance between each bolus. At the end of ET-1 delivery at each site, the needle was left in place for 5 min to allow ET-1 solution diffuse away from the needle tip before withdrawal. Then the operative field was washed with saline and the incision was sutured. During surgery, several vital signs were monitored, including blood oxygen saturation level (> 95%), heart rate (150–220/min),respiratory rate (10–40/min),and blood pressure (> 60 mm Hg mean value, and > 90 mm Hg for systolic pressure) by animal multi-parameter monitor (RM400M, Shenzhen Reward Life Science, Shenzhen, China), see Table 2. After operation, the monkeys received antibiotic treatment of penicillin sodium (100,000 U/kg, intramuscular injection daily) for 3 days to prevent infection. Animals were administered regular doses of pain relievers ibuprofen during the recovery period of 7 days following surgery. We observed motor functional deficits after cortical ET-1 injection, such as a significant decline in motor movement following the surgery, suggesting that the ET-1 injection damaged the motor cortex.

**TABLE 2:** Physiological parameters under anesthesia.

Animals	SaO_2_	Pulse Rate (min./max. beats/min)	Respiratory Rate (min./max. breaths/min)	SAP/DAP (min./max. mmHg)
				
04339#	>95%	146–181	15–22	126–137/76–84
04013#	>95%	153–175	18–25	115–131/72–79
07057#	>95%	151–180	13–21	120–129/76–87
07041#	>95%	148–169	15–22	111–136/73–86
03013#	>95%	143–157	17–25	114–119/62–71
01041#	>95%	146–161	17–37	118–132/79–83
060107#	>95%	137–146	22–25	131–136/72–80
03039#	>95%	143–158	17–24	121–132/59–64
04003#	>95%	136–148	16–21	138–149/69–81
04041#	>95%	148–152	14–18	123–142/80–85
07059#	>95%	129–165	22–40	113–134/67–82
01323#	>95%	132–146	17–22	121–136/76–87
03021#	>95%	145–159	16–18	119–135/73–82
01073#	>95%	139–146	17–22	126–134/60–65
05025#	>95%	129–163	19–22	127–134/67–83
07089#	>95%	145–156	10–16	115–131/60–72
04307#	>95%	164–220	14–31	109–128/68–79
96079#	>95%	147–164	17–32	102–111/61–73

*systolic arterial pressure (SAP), diastolic arterial pressure (DAP).*

### Plasmid Construction and AAV Virus Production

To produce adeno-associated virus (AAV) vectors, ND1-P2A-mCherry was inserted into pAAV-FLEX-GFP vector (Addgene) to replace GFP to generate pAAV-Flex-CAG-ND1-P2A- mCherry. mCherry was inserted into pAAV-FLEX-GFP to replace GFP and was used as a negative control. Human GFAP promoter (1.6 kb) was inserted into pAAV-MCS (Cell Biolabs) to replace CMV promoter, then Cre was subcloned into pAAV-MCS to generate pAAV-hGFAP-Cre. The backbone of pAAV-GFAP::ND1-GFP and pAAV-GFAP::GFP vectors is pAAV-MCS (Cell Biolabs, Inc.). CMV promoter was changed to GFAP promoter, and GFP or NeuroD1-GFP was inserted to MCS. Recombinant AAV9 was produced by HEK293T cell line. The purification method used Iodixanol Gradient Solutions (D1556, Optiprep, Sigma) and desalting and concentrating on Merck Amicon Ultra-15 Centrifugal Filters (UFC910008, Millipore). Purified AAV viruses were titered using QuickTiter AAV Quantitation Kit (VPK-145, Cell biolabs).

### AAV Virus Injection

Animals were anesthetized as mentioned above before fastened to the stereotaxic instrument. Total 10 μl viruses per site were injected using the Hamilton syringe and infusion pump at a speed of 400 nl per minute. AAV viruses were injected in the same sites of ET-1 injection. For brain repair, the AAV virus was injected at 10 days (3 monkeys), 21 days (the majority of the work, 10 monkeys), and 30 days (3 monkeys) post ischemic injury. To test conversion, AAV viruses were directly injected in non-stroke monkey cortex (monkey ID #04339). Virus stocks were diluted to 10^12^ GC/ml for injection. After injection, the needle was kept in place for five additional minutes and then slowly withdrawn. Then, the wound was cleaned thoroughly and sutured. After surgery, monkeys were given penicillin daily for 3 days to prevent infection.

### MRI Scanning

All magnetic resonance imaging (MRI) scans were performed using the LianYing uMR770 3.0-T scanner. Two monkeys were arranged for MRI analysis 1 year after virus injection. Prior to the MRI scan, monkeys were anesthetized intramuscularly as previously described. T2-weighted imaging was acquired in the axial plane and included the following sequences: repetition time (TR) = 2300.0 ms; echo time (TE) = 382.7 ms; field of view (FOV) = 120 X 120 mm (T1), 240 X 240 mm (T2); Matrix = 240 X 240 (T1), 320 X 320 (T2); slice thickness 0.5 mm, no gap.

### Immunohistochemistry

For brain section staining, the monkeys were deeply anesthetized with sodium pentobarbital and perfused transcardially with phosphate-buffered saline (PBS, pH 7.4) followed by 4% (W/V) paraformaldehyde (PFA) in phosphate-buffered saline to fix the brain. Brains were carefully removed from the skull and post fixed in 4% PFA for further 3 days at 4°C, and then cut in 40 μm thickness by a vibratome (VT-1000S; Leica).

For immunofluorescence, coronal brain sections were first pretreated in 0.3% Triton X-100 in PBS for 30 min, followed by incubation in 5% bovine serum albumin (BSA) and 0.1% Triton X-100 in PBS for 1 h at room temperature. For double or triple immunofluorescence, primary antibodies were simultaneously incubated overnight at 4°C in 1% BSA and 0.1% Triton X-100 in PBS. Primary antibodies were used as follows: mouse anti-GFAP (1:1000, SMI-21R, Biolegend), goat anti-NeuroD1 (1:1000, sc-1084, SantaCruz), rabbit anti-NeuN (1:1000, ABN78, Millipore), mouse anti-NeuN (1:500, MAB377, Millipore), mouse anti-MAP2 (1:500, M4403, Sigma), chicken anti-GFAP (1:1000, AB5541 Millipore), rabbit anti-Iba1 (1:1000, 019-19741, Wako), rabbit anti-AQP4 (1:500, AB3594, Millipore), rabbit anti-Parvalbumin (1:1000, ab11427, Abcam), rabbit anti-GFP (1:1000, sc-8334, SantaCruz), chicken anti-GFP (1:1000, ab13970, Abcam), rabbit anti-mCherry (1:1000, ab167453, Abcam), chicken anti-mCherry (1:500, 205402, Abcam), mouse anti-SV2 (1:1000, SV2, DSHB), rabbit anti-Tbr1 (1:500, ad31940, abcam). The next day, primary antibodies were washed off and proper fluorophore-conjugated secondary antibodies were used, Cy2-, Cy3-, and Cy5-conjugated secondary antibodies were obtained from Jackson ImmunoResearch (1:500). Alexa Fluor 488-, Alexa Fluor 594-, and Alexa Fluor 647-conjugated secondary antibodies were obtained from Thermo Fisher Scientific (1:1000). After additional washing in PBS, sections were counterstained with DAPI (4,6-diamidino-2-phenylindole, D8200, Solarbio) in PBS for 10 min. The sections were then rinsed 3 times with PBS and mounted onto a glass slide with a Fluorescent Mounting Media (71-00-16, KPL). Stained sections were examined and photographed with Nikon A1+ or Zeiss LSM880 confocal microscopy. Z-stacks of digital images were acquired using Nikon A1+ or Zeiss LSM880 confocal microscopy.

For DAB staining, endogenous peroxidase activity was quenched through incubation of sections in 3% H_2_O_2_ for 10 min at room temperature prior to washing with PBS, and sequential incubations with blocking serum and primary antibodies (overnight 4°C) were performed. Secondary peroxidase-labeled affinity purified goat anti-mouse (474-1806, KPL) and goat anti-rabbit antibodies (474-1516, KPL) were used at 1/500 dilution for 2 h. After washing in PBS, the sections were developed with diaminobenzidine (DAB). The sections were rinsed with PBS and then mounted on slides, dehydrated, and coversliped. Sections were viewed with microscope (Olympus, CX41; camera: Olympus DP25; software: CellSens Entry 1.4.1; Japan).

### Statistical Analyses

All images for quantitative analyses were acquired by a confocal microscope (Zeiss LSM880, Jena, Germany). Data was presented as mean ± SEM or mean ± SD. Shapiro-Wilk normality test and Kolmogorov-Smirnov normality test were used for Normality test. Mann–Whitney test and Wilcoxon matched-pairs signed rank test were applied using GraphPad Prism v8.00 (GraphPad Software, La Jolla, CA, United States). Histological tests and quantification were analyzed blindly.

## Data Availability Statement

The raw data supporting the conclusions of this article will be made available by the authors, without undue reservation.

## Ethics Statement

The animal study was reviewed and approved by The Ethics Committee of Kunming Institute of Zoology and The Kunming Primate Research Center.

## Author Contributions

GC conceived and supervised the entire project, analyzed the data, and wrote the manuscript. L-JG performed the majority of the experiments with significant help from F-HY analyzed the data, and participated in writing the manuscript. L-JG and F-HY performed monkey brain surgery experiments with the assistance of N-HC, YL, and JF. L-JG made viruses and performed the MRI experiment. L-JG, F-HY, MJ, and JF all contributed to immunostaining experiment. WL and TW performed Brdu experiments in mouse stroke model. X-TH and J-HW participated in the discussion of the experiments. All authors contributed to the article and approved the submitted version.

## Conflict of Interest

GC is a co-founder of NeuExcell Therapeutics Inc. The remaining authors declare that the research was conducted in the absence of any commercial or financial relationships that could be construed as a potential conflict of interest.
