# Supplementary material for: In vivo Neuroregeneration to Treat Ischemic Stroke Through NeuroD1 AAV-Based Gene Therapy in Adult Non-human Primates
Source: Front Cell Dev Biol. 2020 Nov 5;8:590008. doi: 10.3389/fcell.2020.590008 (PMC7674285; doi:10.3389/fcell.2020.590008)
Supplement: Supplementary file 1 [file Table_1.DOCX]

**SUPPLEMENTARY MATERIAL**

***In Vivo* Neuroregeneration to Treat Ischemic Stroke in Adult Non-Human Primate Brains through NeuroD1 AAV-based Gene Therapy**

Long-Jiao Ge^1,7,^ *^, †^, Fu-Han Yang^1,^ *, Wen Li^2^, Tao Wang^2^, Yu Lin^6^, Jie Feng^1^, Nan-Hui Chen^1^, Min Jiang^4^, Jian-Hong Wang^6, †^, Xin-Tian Hu^1, 5, 6†^, Gong Chen^1,2,3, †^

**Supplementary Figures and Legends:**

**
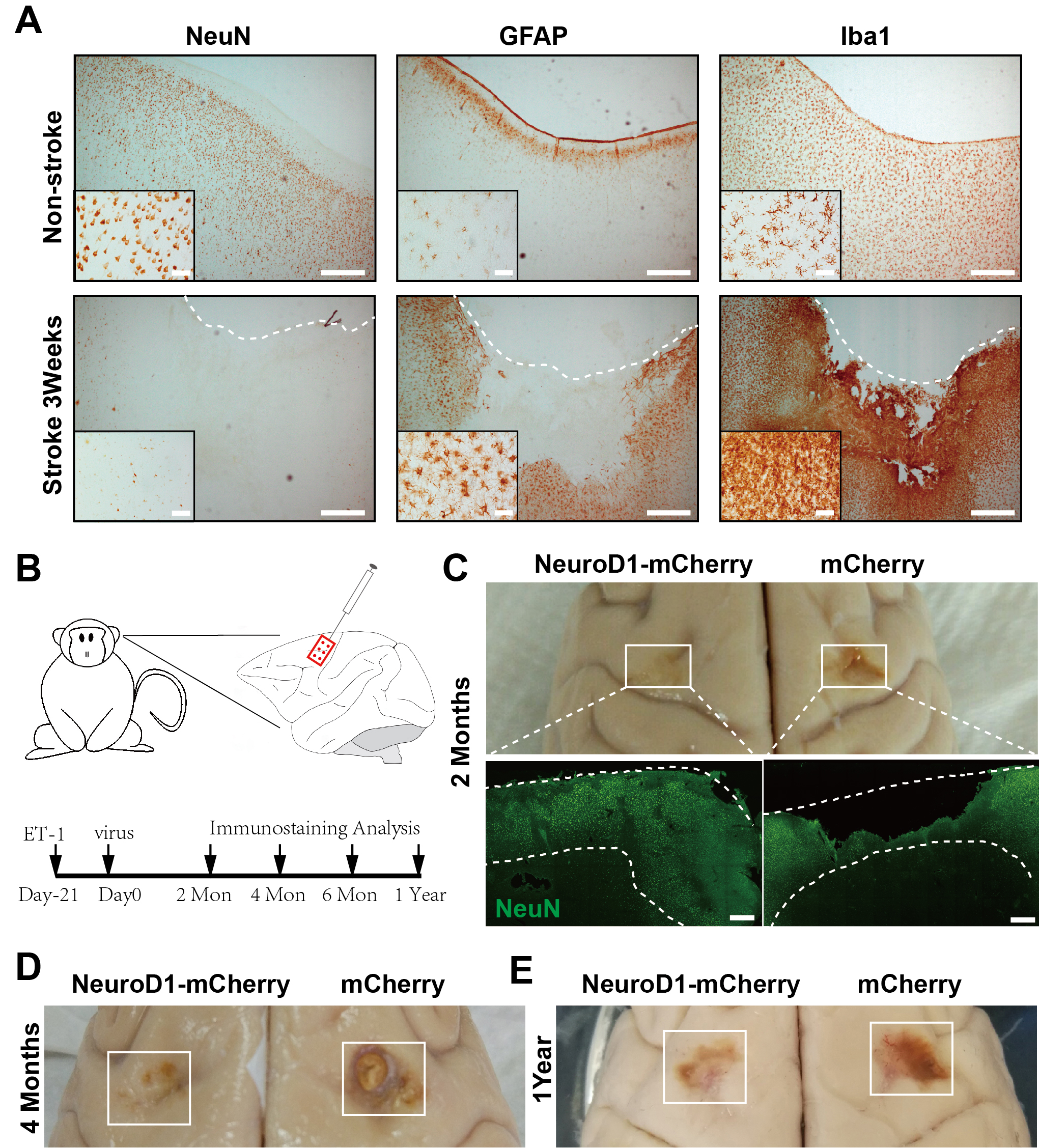
**

**Figure S1. Establishment of NHP ischemic cortical stroke model and brain repair by NeuroD1 AAV-based gene therapy.**

**(A)** Representative images showing NeuN, GFAP and Iba1 immunostaining from cortical sections of non-stroke cortex (top row) and the endothelin-1 (ET-1, 1-31) induced ischemic stroke cortex (bottom row). Note that loss of neurons and gliosis occurred at 3 weeks after focal ischemic injury. Scale bars, 500 μm (low mag), 50 μm (high mag).

**(B)** Schematic diagram illustrating experimental design for the majority of NeuroD1 gene therapy intervention.

**(C)** Representative images showing brain tissue integrity at 2 months following viral injection after stroke. Note a significant tissue damage in the mCherry-injected side (top right) compared to the NeuroD1-injected side (top left), which was further confirmed by immunostaining of NeuN (green) (bottom row). Dashed lines indicate cortical areas. Scale bars, 1000 μm.

**(D-E)** Representative images illustrate less tissue damage in NeuroD1-treated side compared to control mCherry-treated side at 4 months (D) and 1 year (E) following viral injection after stroke.


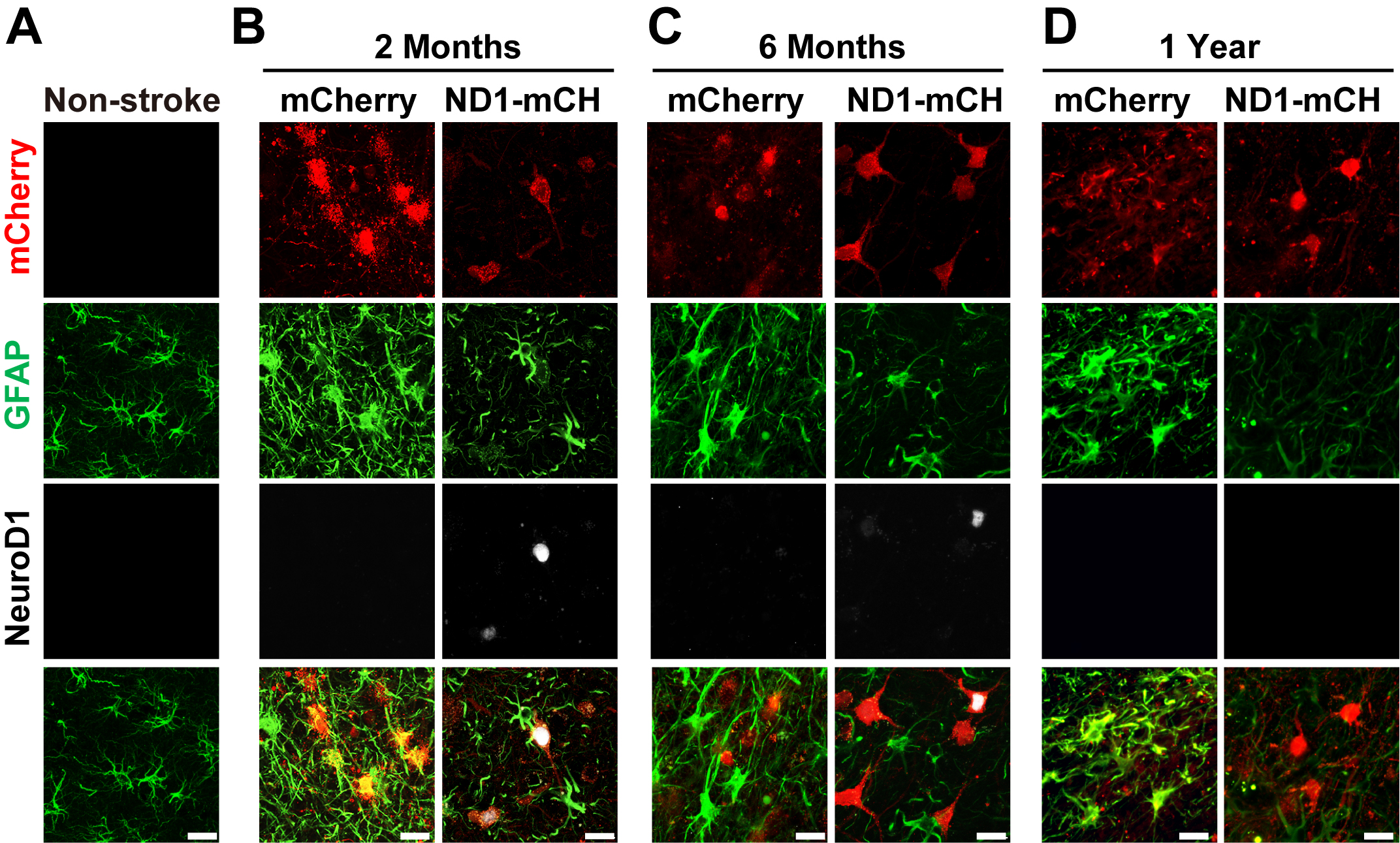


**Figure S2. Astrocytes are not depleted after NeuroD1-mediated conversion.**

**(A-D)** Representative images showing triple immunostaining of mCherry (red), GFAP (green), and NeuroD1 (white) in brain sections obtained from non-stroke monkey (A) and stroke monkeys at 2 months (B), 6months (C), and 1 year (D) after viral infection. Note that in NeuroD1-infected areas, astrocytes were always present and their morphology was less reactive, accompanied with less GFAP protein level compared to the control mCherry-infected cortex. Scar bars, 20 μm.

**
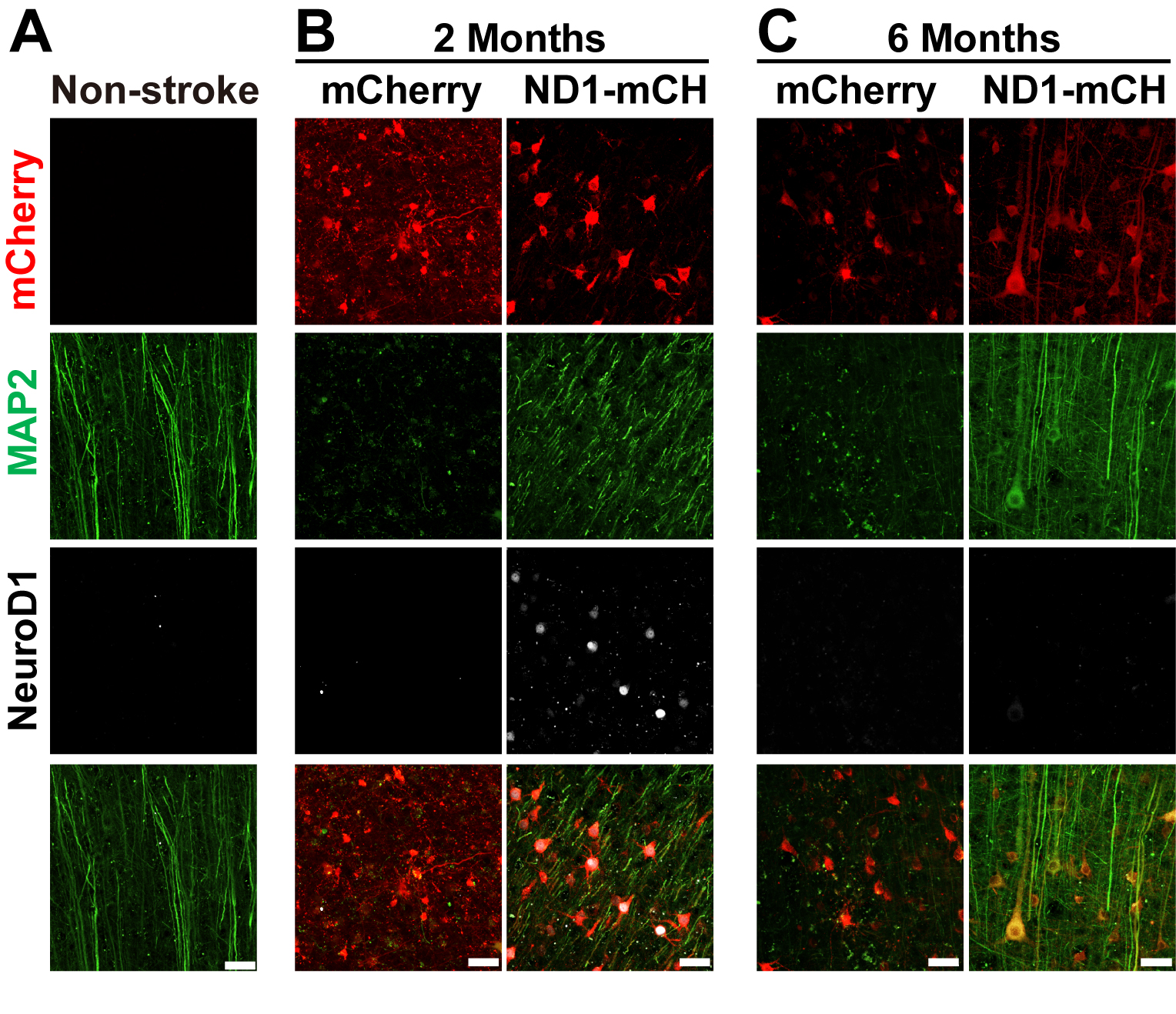
**

**Figure S3. NeuroD1-treatment rescued neuronal dendritic morphology after ischemic stroke in monkey cortex.**

**(A-C)** Representative images showing triple immunostaining of mCherry (red), MAP2 (green) and NeuroD1 (white) in brain sections obtained from non-stroke cortex (A) and ischemic injured cortex at 2 months (B) and 6 months (C) after viral injection. Note that NeuroD1 expression at 6 months after viral infection was significantly reduced, suggesting a potential self-downregulation mechanism. Neuronal dendrites labeled by MAP2 were significantly rescued in the NeuroD1 group compared to the control group. Scar bars, 50 μm.


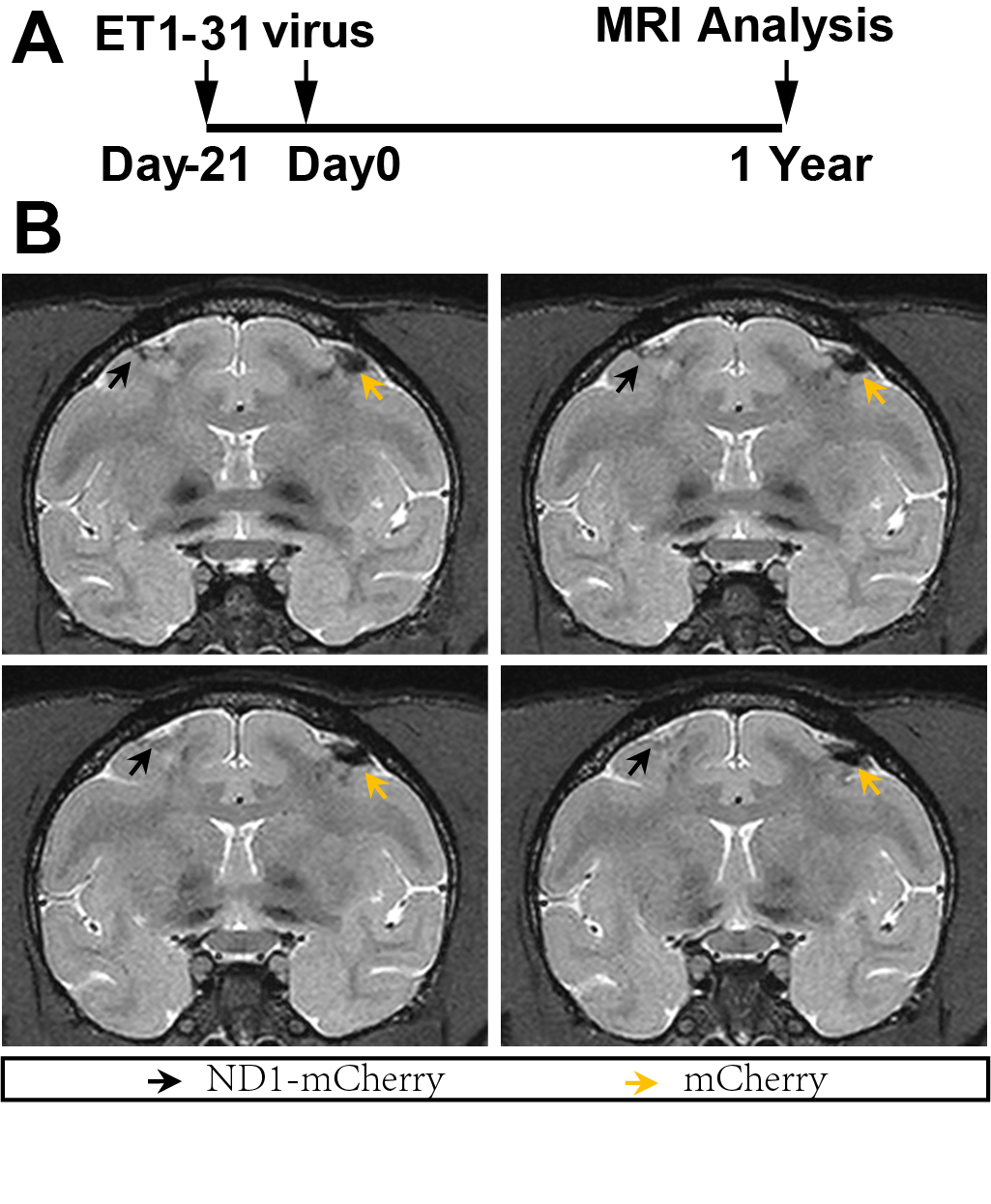


**Figure S4. Brain tissue repair of monkey cortex by NeuroD1-treatment revealed by T2-weighted magnetic resonance imaging (MRI) after ischemic injury.**

**(A)** Timeline for experimental design.

**(B)** T2-weighted MRI images of a monkey brain showing ischemic injured motor cortex at 1 year after AAV viral infection in the control side (right，yellow arrow) and NeuroD1 treatment side (left，black arrow). Arrow points to the location of lesion.


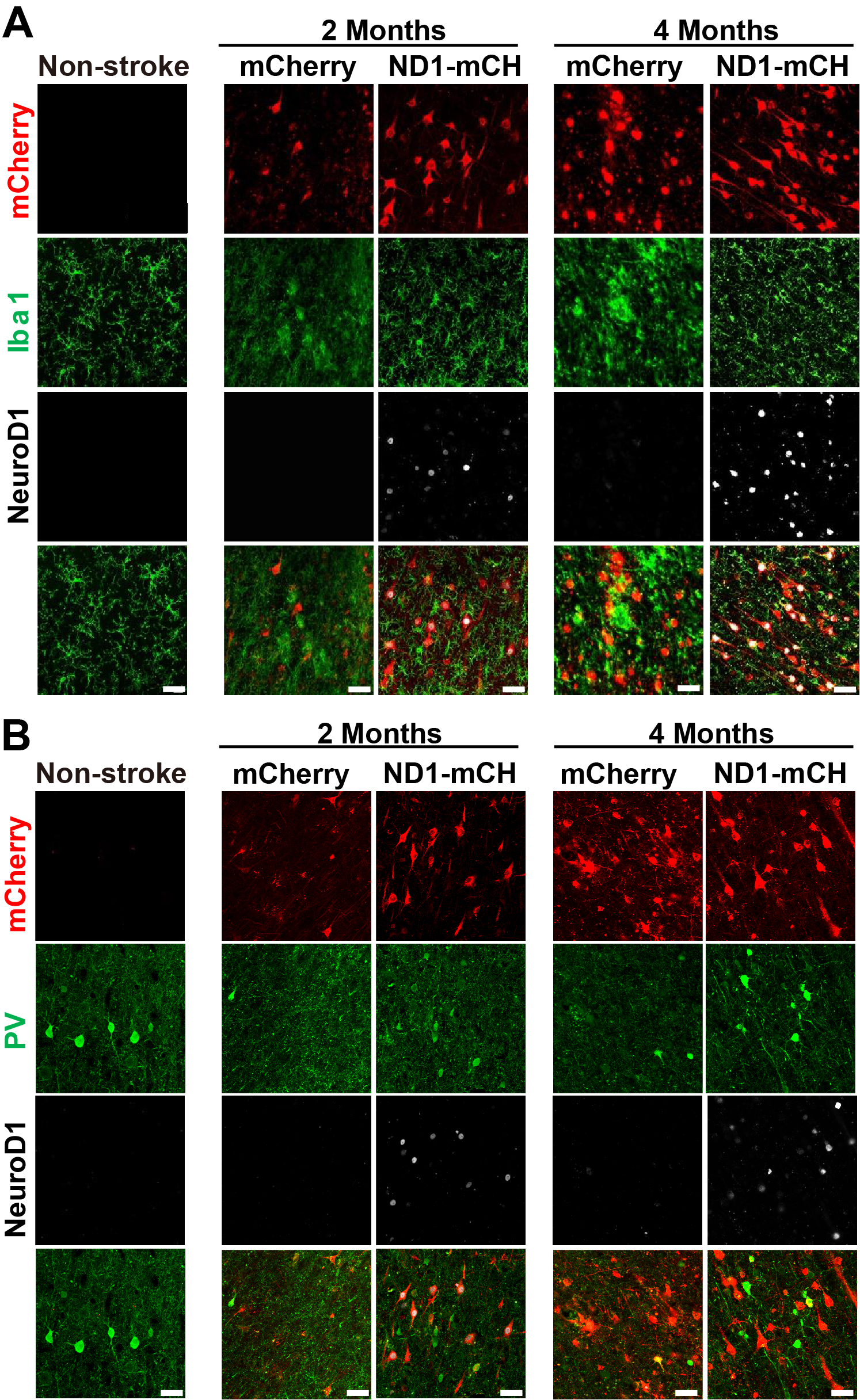


**Figure S5. NeuroD1-treatment reduces microglia and macrophage and promotes PV^+^ interneuron survival.**

**(A)** Representative images showing mCherry (red), Iba1 (green) and NeuroD1 (white) expression pattern in non-stroke cortex (left column) or ischemic cortex (right 4 columns) with virus injection at 10 days post stroke. Note that microglia and macrophage (Iba1) showed less reactive morphology in NeuroD1-infected areas. Scar bars, 50 μm.

**(B)** Representative images showing mCherry (red), PV (green) and NeuroD1 (white) expression pattern in non-stroke cortex (left column) or ischemic cortex (right 4 columns). AAV infection at 10 days post stroke. Note a significant rescue of PV^+^ interneurons in NeuroD1-infected areas. Scar bars, 50 μm.


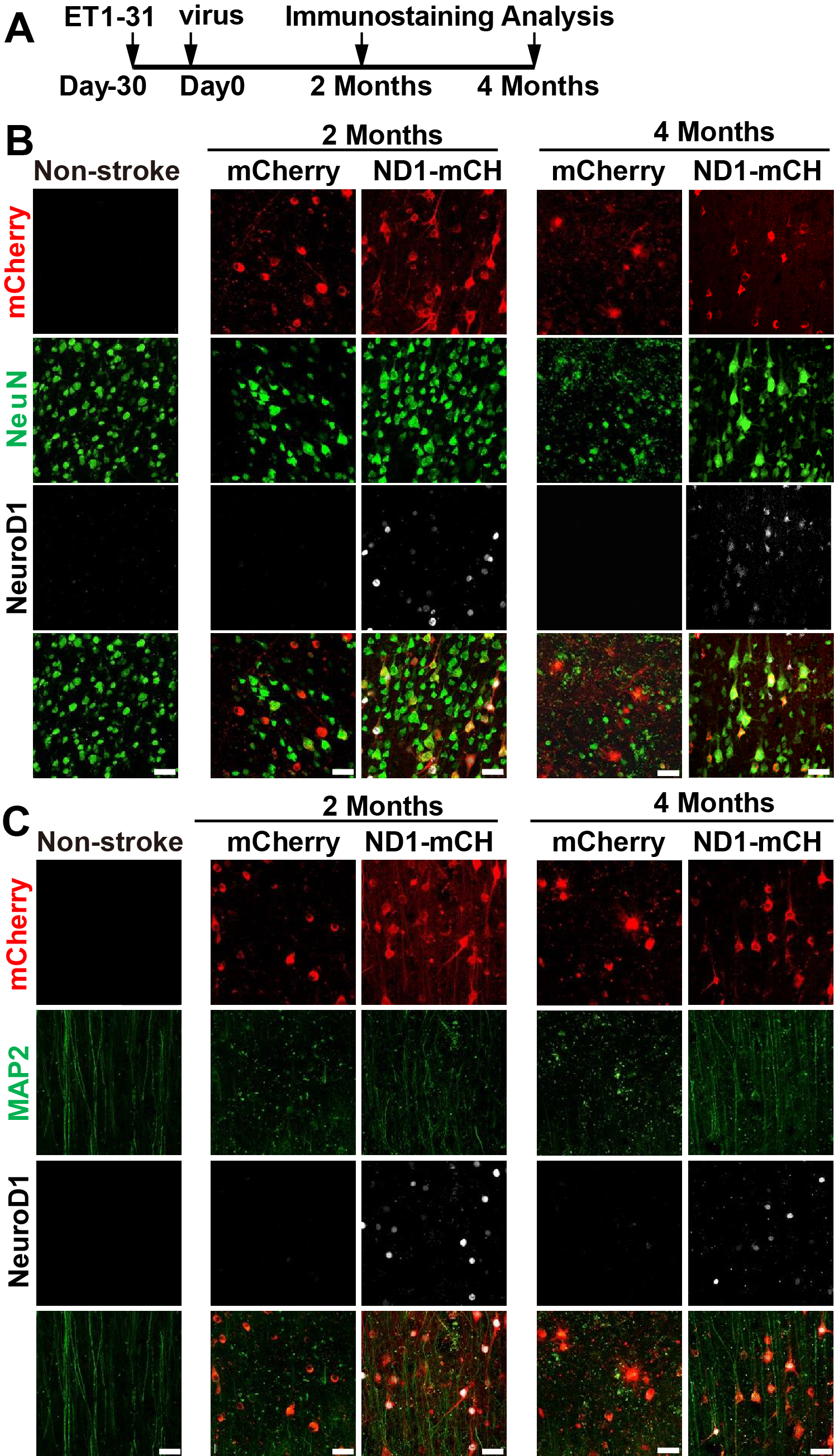


**Figure S6. NeuroD1-treatment at 30 days after ischemic stroke still rescued neuronal density in monkey cortex.**

**(A)** Timeline showing experimental design.

**(B)** Representative images showing mCherry (red), NeuN (green) and NeuroD1 (white) expression pattern in non-stroke cortex (left column) and ischemic cortex (right 4 columns) with virus infection at 30 days post stroke. Scar bars, 20 μm.

**(C)** Representative images showing mCherry (red), MAP2 (green) and NeuroD1 (white) expression pattern without (left column) or with stroke (right 4 columns). Scar bars, 20 μm.


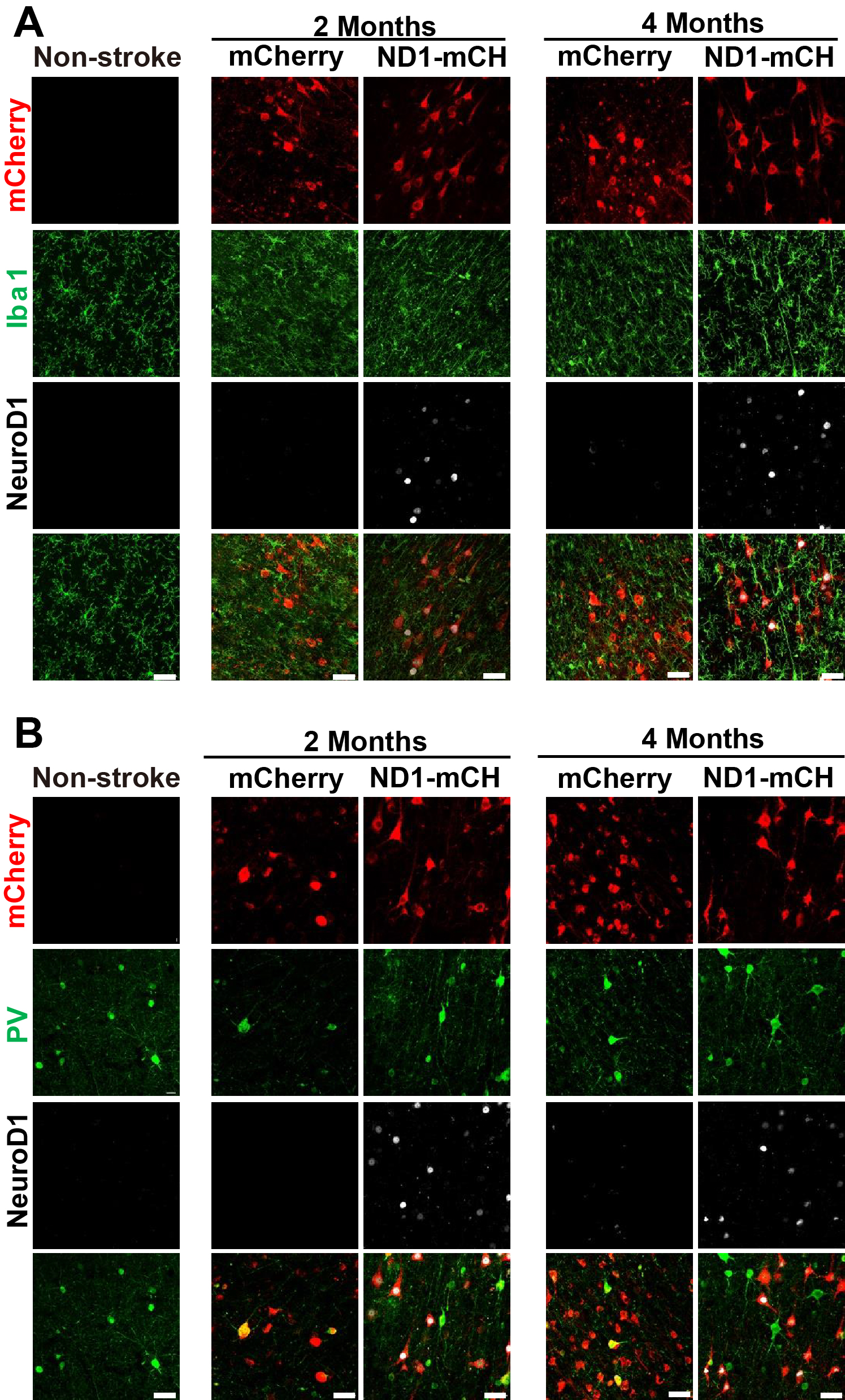


**Figure S7. NeuroD1-treatment at 30 days after ischemic stroke reduces microglia and macrophage and promotes PV^+^ neuron survival.**

**(A)** Representative images showing mCherry (red), Iba1 (green) and NeuroD1 (white) expression pattern in non-stroke cortex (left column) and ischemic cortex (right 4 columns). Virus infection at 30 days post stroke. Scar bars, 50 μm.

**(B)** Representative images showing mCherry (red), PV (green) and NeuroD1 (white) expression pattern in non-stroke cortex (left column) and ischemic cortex (right 4 columns) with virus infection at 30 days post stroke. Note that PV neurons were rescued in NeuroD1-infected areas. Scar bars, 50 μm.


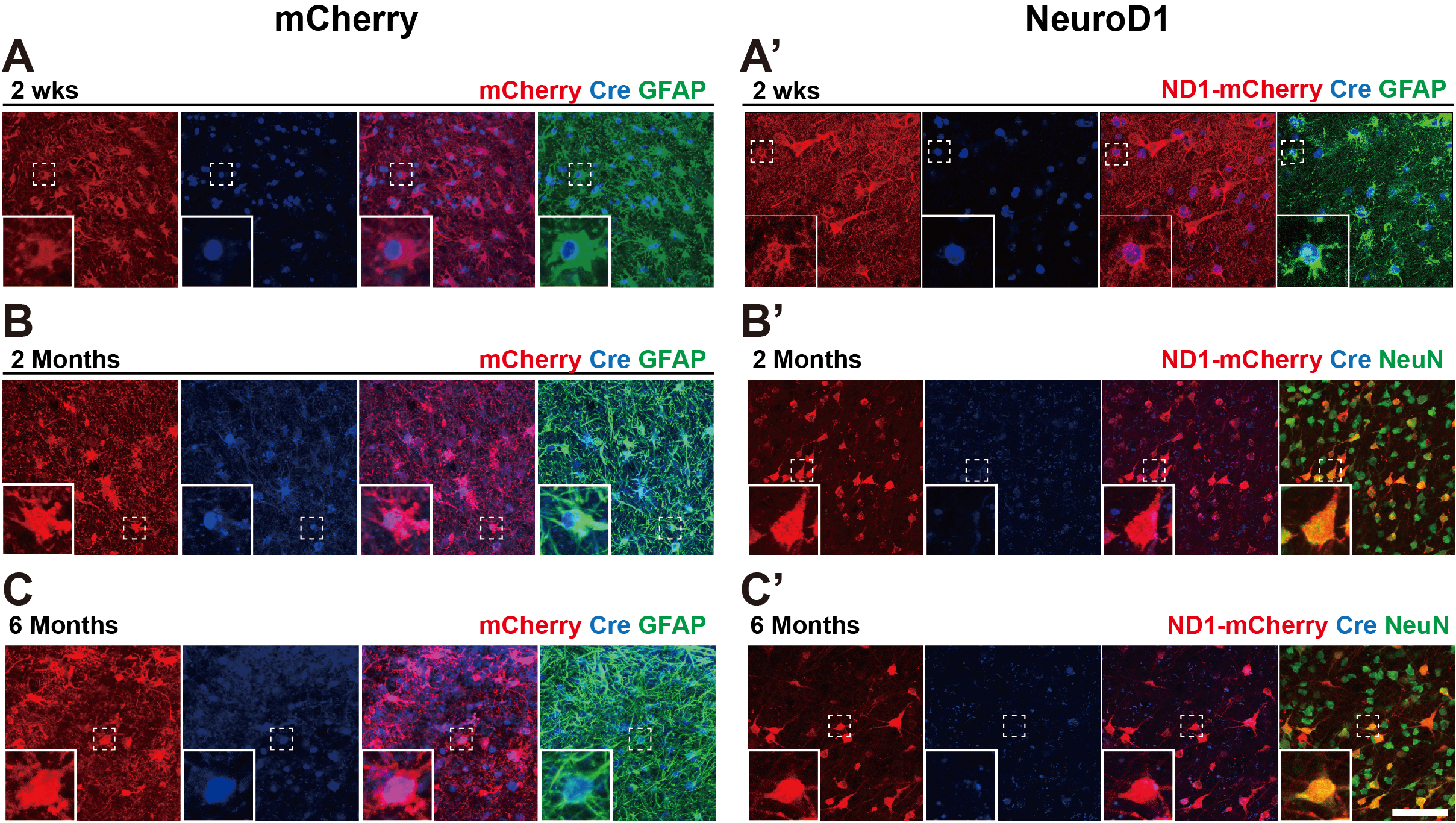


**Figure S8. Comparison of the Cre signal in the motor cortex of monkey between the control (left) and NeuroD1 groups (right) after stroke.**

(A, B, C) Cre staining in the control side infected by mCherry alone.

(A’, B’, C’) Cre staining in the NeuroD1-side.

Note that in the control mCherry side, Cre expression was localized in astrocytes labeled by GFAP from 2 weeks to 6 months after viral infection.

In the NeuroD1-infected side, however, Cre was only detected in astrocytes labeled by GFAP at 2 weeks of viral infection (AAV9-GFAP-Cre + AAV9-Flex-D1-mcherry), but not at 2 or 6 months following NeuroD1-viral injection, when most NeuroD1-infected astrocytes were already converted into NeuN^+^ neurons (yellow). Scale bars, 50 µm.


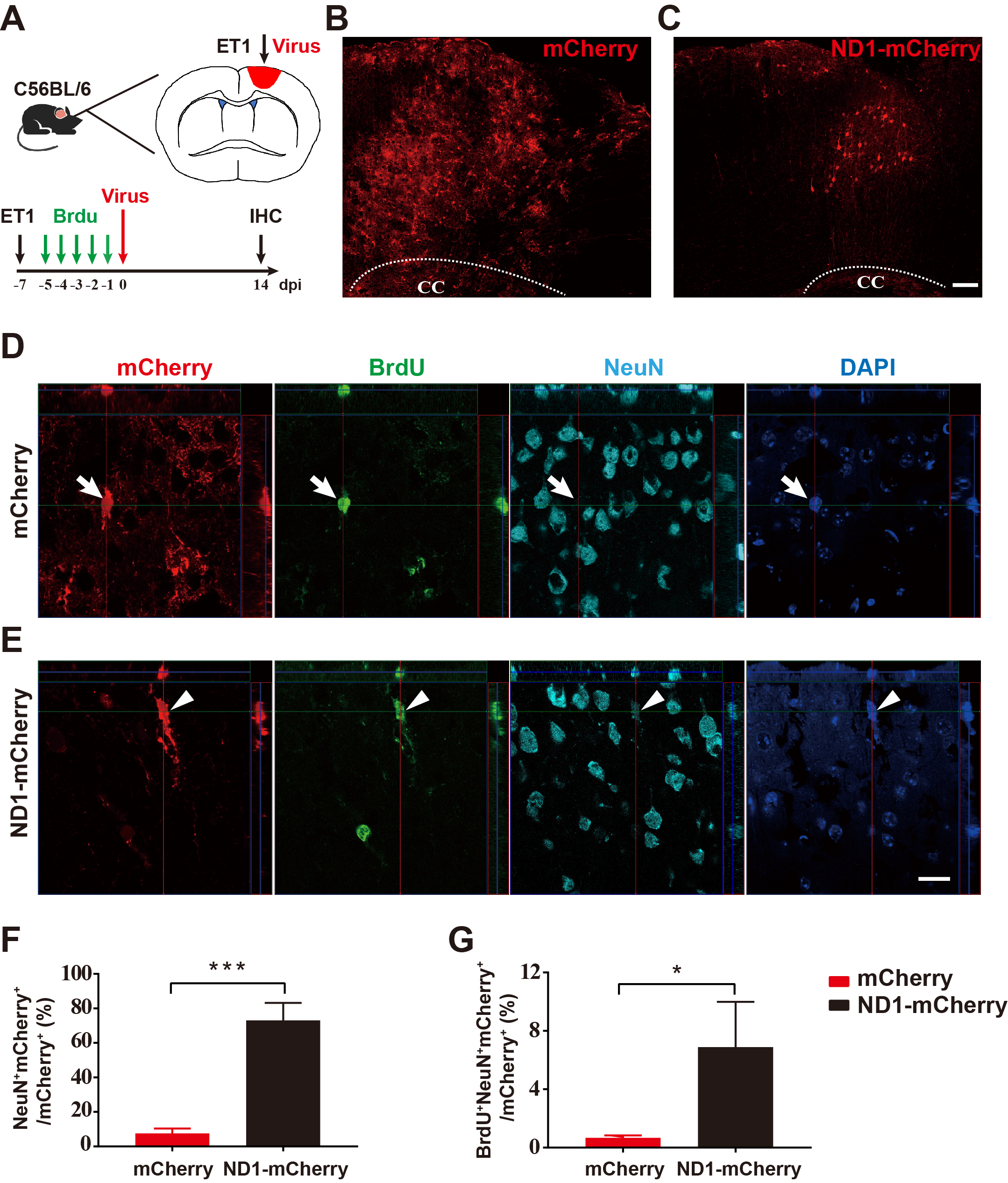


**Figure S9. Converted neurons were derived from mouse proliferative astrocytes.**

**(A)** Experimental design. ET-1, endothelin-1. DPI, days post injection. ND1, NeuroD1.

**(B-C)** Representative pictures showing the infection area and cell morphology of the control (**B**) and NeuroD1 (**C**) group at 14 days post viral infection. The white dashed line outlines the corpus callosum. CC, corpus callosum. Scar bar, 100 μm.

**(D-E)** Representative pictures showing BrdU positive (green) signal among the viral infected cells (mCherry positive, red). In the control group (**D**), BrdU-labeled cell was negative for NeuN (cyan), but some BrdU-labeled cell was found positive for NeuN (cyan) in the NeuroD1 group (**E**). White arrows and arrow heads indicate the co-labeled cells in control and NeuroD1 group, respectively. Scar bar, 25 μm.

**(F)** Quantification of NeuN and mCherry-double positive cells in the control mCherry and NeuroD1-mCherry group.

**(G)** Quantification of NeuN, mCherry and BrdU-triple positive cells in the control and NeuroD1 group. Cell number quantified in the experiment: the control group, 326.7 ± 50.0 cells/animal, animal number = 3; NeuroD1 group, 246.7 ± 64.0 cells/animal, animal number = 3. *** *P = 0.0004*, * *P = 0.025*, unpaired 2-talied *t* test.
